# Chemical Genetic Validation of CSNK2 Substrates Using an Inhibitor-Resistant Mutant in Combination with Triple SILAC Quantitative Phosphoproteomics

**DOI:** 10.3389/fmolb.2022.909711

**Published:** 2022-06-09

**Authors:** Laszlo Gyenis, Daniel Menyhart, Edward S. Cruise, Kristina Jurcic, Scott E. Roffey, Darren B. Chai, Flaviu Trifoi, Sam R. Fess, Paul J. Desormeaux, Teresa Núñez de Villavicencio Díaz, Adam J. Rabalski, Stephanie A. Zukowski, Jacob P. Turowec, Paula Pittock, Gilles Lajoie, David W. Litchfield

**Affiliations:** ^1^ Department of Biochemistry, Schulich School of Medicine & Dentistry, Western University, London, ON, Canada; ^2^ Department of Oncology, Schulich School of Medicine & Dentistry, Western University, London, ON, Canada

**Keywords:** protein kinase CK2, CSNK2, chemical genetics, CX-4945, phosphoproteomics, SILAC, mass spectrometry, kinase-substrate relationship validation

## Abstract

Casein Kinase 2 (CSNK2) is an extremely pleiotropic, ubiquitously expressed protein kinase involved in the regulation of numerous key biological processes. Mapping the CSNK2-dependent phosphoproteome is necessary for better characterization of its fundamental role in cellular signalling. While ATP-competitive inhibitors have enabled the identification of many putative kinase substrates, compounds targeting the highly conserved ATP-binding pocket often exhibit off-target effects limiting their utility for definitive kinase-substrate assignment. To overcome this limitation, we devised a strategy combining chemical genetics and quantitative phosphoproteomics to identify and validate CSNK2 substrates. We engineered U2OS cells expressing exogenous wild type CSNK2A1 (WT) or a triple mutant (TM, V66A/H160D/I174A) with substitutions at residues important for inhibitor binding. These cells were treated with CX-4945, a clinical-stage inhibitor of CSNK2, and analyzed using large-scale triple SILAC (Stable Isotope Labelling of Amino Acids in Cell Culture) quantitative phosphoproteomics. In contrast to wild-type CSNK2A1, CSNK2A1-TM retained activity in the presence of CX-4945 enabling identification and validation of several CSNK2 substrates on the basis of their increased phosphorylation in cells expressing CSNK2A1-TM. Based on high conservation within the kinase family, we expect that this strategy can be broadly adapted for identification of other kinase-substrate relationships.

## Introduction

The coordinated phosphorylation of serine, threonine, and tyrosine residues at distinct sites within proteins enables intricate control of cellular processes (Cohen, 2002). It is estimated that up to 90% of all proteins are phosphorylated (Wilson et al., 2018). Despite an increasing wealth of information regarding phosphorylated sites in living cells, the majority have yet to be attributed to any one of the more than 500 kinases encoded in the genome (Manning et al., 2002; Lemeer and Heck, 2009; Wilson et al., 2018). The lack of knowledge surrounding kinase-substrate relationships prevents a thorough understanding of complex signalling networks and biological processes in cells.

Casein Kinase 2 (CSNK2) is a ubiquitously expressed protein kinase with an integral role in the regulation of key cellular processes (Litchfield and Gyenis, 2015; Rabalski et al., 2016). These include cell cycle progression (Litchfield and Lüscher, 1993; St-Denis et al., 2009; St-Denis et al., 2011), transcription (Cabrejos et al., 2004), circadian rhythms (Tsuchiya et al., 2009), apoptosis (Ahmad et al., 2008; Wang et al., 2008; Duncan et al., 2010; Duncan et al., 2011; Turowec et al., 2011; Turowec et al., 2013; Turowec et al., 2014), and DNA repair (Loizou et al., 2004; Ali et al., 2009; Xu et al., 2014). With approximately 20% of the known phosphoproteome adhering to its [S/T]-X-X-[D/E] minimal recognition motif (Meggio and Pinna, 2003; Salvi et al., 2009), CSNK2 is suggested to be one of the most pleiotropic human kinases. The CSNK2-dependent phosphoproteome is likely even more expansive when considering the ability of CSNK2 to phosphorylate outside of this recognition motif (Meek et al., 1990). In mammalian cells, CSNK2 exists primarily as a tetrameric holoenzyme composed of two catalytic alpha (CSNK2A1, UniProt: P68400) and/or alpha’ (CSNK2A2, UniProt: P19784) subunits complexed with two regulatory beta subunits (CSNK2B, UniProt: P67870). However, the catalytic CSNK2A1 and CSNK2A2 subunits can also function independently of the regulatory CSNK2B subunits (Litchfield, 2003). Deletion of either the CSNK2A1 or CSNK2B subunits results in an embryonic lethal phenotype in mice indicating that CSNK2 is essential for organismal development. Due to its fundamental role in regulating cellular processes, CSNK2 is also implicated in neurological disease (e.g., Alzheimers) and numerous malignancies when dysregulated (Rosenberger et al., 2016; Castello et al., 2017; Chua et al., 2017; Strum et al., 2021).

Mapping the CSNK2-dependent phosphoproteome is necessary for better characterization of its central role in cellular signalling. An increased understanding of the physiological signalling coordinated by CSNK2 will further reveal how perturbation of these pathways is implicated in disease. While several studies have attempted direct, large-scale CSNK2 substrate identification, they have done so either *in vitro* or in cell lysate. *In vitro* studies which attempt to determine *bona fide* CSNK2 substrates using purified components do not fully recapitulate kinase activity and substrate specificity in living cells, which is not only dictated by the recognition motif of substrates, but also by subcellular localization, scaffolding/adaptor protein interactions, and post-translational modification of substrates and CSNK2 itself (Pinna and Ruzzene, 1996; Good et al., 2011; Roffey and Litchfield, 2021). Although CSNK2 substrate discovery experiments performed in lysate offer some improvement over those done *in vitro*, they are unable to capture the spatiotemporal context of CSNK2 activity in living, intact cells.

Indirect methods of kinase-substrate identification better reflect the spatiotemporal context of kinase activity in living cells and may therefore be more likely to identify true cellular substrates. For example, a chemical genetics approach in which cells are treated with a membrane-permeable kinase inhibitor is most often utilized. Those sites which demonstrate reduced phosphorylation relative to control cells not treated with inhibitor are presumed to be substrates. Combination of this method with quantitative phosphoproteomic analysis enables the large-scale identification of putative substrates (Shah and Kim, 2019; Jurcik et al., 2020). The success of this approach relies mainly on the availability of specific, potent inhibitors for the kinase of interest. Due to the central role of protein kinase CSNK2 in multiple cellular processes and malignancies (Chua et al., 2017; Strum et al., 2021), several CSNK2-directed inhibitors have been developed (Sarno et al., 2001; Pagano et al., 2004a; Pagano et al., 2004b; Cozza et al., 2006; Perea et al., 2008; Cozza et al., 2009; Sandholt et al., 2009; Siddiqui-Jain et al., 2010; Hou et al., 2012). However, these CSNK2-directed compounds exhibit variable specificity and selectivity towards their intended kinase target—this is especially true of compounds targeting the highly conserved ATP-binding site (Duncan et al., 2008; Gyenis et al., 2011; Duncan et al., 2012; Gyenis et al., 2013a; Gyenis et al., 2013b). Consequently, the differential phosphorylation of several phosphosites may be incorrectly attributed to CSNK2 activity when they are in fact due to off-target effects of the inhibitor. Moreover, CSNK2-directed inhibitors exhibit variable potency in living cells, which is not only dependent on their ability to bind CSNK2 and effectively compete with ATP and GTP, but also their ability to cross cellular and internal membranes. To achieve a sufficient level of CSNK2 inhibition, inhibitors of low potency must be utilized at high concentrations, which results in further decreased specificity (Knight and Shokat, 2005).

To overcome limitations noted above, we devised an approach for the large-scale identification and validation of CSNK2 substrates in living cells. The use of an inhibitor-resistant mutant kinase can clarify differential phosphorylation resulting from on- versus off-target effects of inhibitors. The mutant kinase remains active in the presence of the inhibitor and therefore, *bona fide* sites remain phosphorylated when compared with the inhibitor-sensitive wild-type kinase. Phosphosites which are downregulated due to off-target effects remain downregulated in cells expressing the inhibitor-resistant mutant. Previously, we had developed a Flp-In T-REx osteosarcoma (U2OS) cell line expressing a double mutant of CSNK2A1 (DM, V66A/I174A) under tetracycline control, a CSNK2 mutant which resisted inhibition by TBB and derivatives. This inhibitor-resistant mutant was instrumental in identifying and validating eukaryotic elongation factor 1 delta (EEF1D) pS162 as a *bona fide* CSNK2 phosphosite substrate (Gyenis et al., 2011). We have since developed a Flp-In T-REx U2OS cell line expressing a triple mutant of CSNK2A1 (TM, V66A/H160D/I174A) under tetracycline control. When compared with DM-CSNK2A1, TM-CSNKA1 better resists inhibition of catalytic activity by CX-4945, a clinical stage CSNK2-directed ATP-competitive inhibitor. Therefore, we exploited this TM-CSNK2A1 in combination with a triple SILAC-based (Stable Isotope Labelling of Amino Acids in Cell Culture) quantitative phosphoproteomics strategy for the systematic identification of *bona fide* CSNK2 substrates in living cells.

## Experimental Procedures

### Reagents, Antibodies, and Immunoblotting

All reagents used in the assays were from Sigma or indicated otherwise. CSNK2 inhibitors tested in this study were TBB (4,5,6,7-Tetrabromobenzotriazole), TBBz (4,5,6,7-Tetrabromobenzimidazole), DMAT (2-Dimethylamino-4,5,6,7-tetrabromo-1H-benzimidazole; Calbiochem), Ellagic acid, Quinalizarin (1,2,5,8-Tetrahydroxy-9,10-anthraquinone; ACP Chemicals, Inc. Canada), Resorufin (7-Hydroxy-3H-phenoxazin-3-one), CSNK2 inhibitor 8 (4-(2-(4-Methoxybenzamido)thiazol-5-yl)benzoic acid), CX-4945 (5-(3-chlorophenylamino)benzo[c][2,6]naphthyridine-8-carboxylic acid; for earlier works synthesized at Department of Pharmacology, University of North Carolina at Chapel Hill; or at MedKoo Biosciences, Inc., Chapel Hill, NC, USA). Immunoblotting was done as described earlier by Gyenis et al. (2013b). Briefly, for the detection of CSNK2A1 and CSNK2A2, polyclonal rabbit antibodies were raised against the C-terminal 51 amino acid sequence of CSNK2A2 (CSK21 and CSK22, 1:2,000; BAbCO, Berkley, CA) that can recognize both catalytic subunits of CSNK2 (Bosc et al., 1995). For detection of total CSK2B (1:2,000) we followed established protocols (Towbin et al., 1992) at the indicated dilutions in 3% BSA in TBST (contains 0.05% Tween 20, used for all rabbit antibodies) or PBST (contains 0.1% Tween 20, used for all mouse antibodies) for primary, and 1% BSA in TBST or PBST for secondary antibodies. For the C-terminal HA-tagged form of CSNK2A1-HA detection the monoclonal anti-HA 3F10 (1:500; Roche) antibody was used. The anti-Glyceraldehyde-3-Phosphate Dehydrogenase (GAPDH), clone 6C5 (1:1,000; Millipore) antibody was used to assess equal loading. We monitored CSNK2-dependent phosphorylation with the following commercially available phospho-specific antibodies: CSNK2 pS/pTDXE motif antibody (1:1,000; Cell Signaling), XRCC1 pS518/T519/T523 (1:2,500; Cell Signaling), CDC37 pS13 (1:25,000; Abcam). Additionally, we raised phosphospecific rabbit antibodies against eukaryotic translation initiation factor 2 subunit beta pS2 (EIF2S2; 1:10,000) using the ac-pS-GDEMIFDPTMSKC-amide peptide; against CSNK2B pS2/3/8 (CSNK2B pS2/3/8; 1:10,000) using ac-pS–pS–pS–EEVSMISWFC-amide mixed with ac-pS–pS–pS–EEV-pS-MISWFC-amide peptides (1:1); against DNA ligase 1 pS36 (LIG1, 1:5,000) using the C-Ahx-KAARVLGpSEGEEED-amide peptide; and against single stranded binding protein SSB pS366 using the C-Ahx-KTKFApSDDEHDEH-amide peptide (1:10,000). We also raised rabbit antibodies against the nonphosphorylated SSB protein site S366 using the C-Ahx-KTKFASDDEHDEH-amide peptide (1:5,000). All antibody production and affinity purification were done as was reported earlier (Gyenis et al., 2011) for eukaryotic elongation factor 1 delta pS162 (EEF1D pS162; 1:20,000) at YenZym Antibodies, LLC, San Francisco, CA following their proprietary company protocols. To visualize and validate the phospho-specificity of our antibodies, we treated cell lysate +/-λ-protein phosphatase (New England Biolabs). We also immunoblotted with total EIF2S2 (1:500, Novus in Figure 4D; or 1:3,000; GeneTex in Supplementary Figure S2A). Infrared IRDye-labeled antibodies (1:10,000; LiCor) were used for immunoblot visualization and densitometry quantification on the LiCor Odyssey Infrared Imaging System with the Odyssey V3.0 software.

Peptide competition assays were performed with the visualization procedure previously stated with the exception that primary antibodies were pre-incubated for 30 min at room temperature with at least 200-fold molar excess of the non-phosphorylated peptide or phosphorylated peptide against which the antibody was raised.

### Cell Culture and Cell Line Development

The human adenocarcinoma HeLa Tet-Off (HeLaT, Clontech) and osteosarcoma U2OS derived cells expressing the tetracycline responsible element (U2OS, gift from Dr. Christoph Englert, Forschungszentrum Karlsruhe, Germany (Englert et al., 1995)), or U2OS cells expressing the tetracycline responsible element of Flp-In™ T-REx system (FT-U2OS, gift from Karmella Haynes, Arizona State University (Haynes and Silver, 2011)) were cultured in Dulbecco’s Modified Eagle’s medium (DMEM, Wisent) supplemented with 10% fetal bovine serum (FBS, HyClone), 100 μg/ml streptomycin and 100 units/mL penicillin (Thermo) at 37 °C with 5% CO_2_ in 10 or 15 cm dishes (TPP, FroggaBio), 6- or 12-well plates (Greiner Bio-One). Following the recommendations of Flp-In™ T-REx cell line development of Thermo Fisher Scientific (www.thermofisher.com) we developed and characterized FT-U2OS cell lines stably expressing the wild type CSNK2A1-WT (C-terminal HA tag) or inhibitor-resistant forms of triple mutant CSNK2A1-TM (V66A/H16D/I174A, C-terminal HA tag) with tight tetracycline regulation.

### Inhibitor Treatment–Evaluation by Immunoblotting

Prior to the inhibitor treatments the U2OS cells were plated at 8 × 10^5^ cells per well in 6-well dishes. The cells were challenged with CSNK2 inhibitors at 80% cell confluency using three different inhibitor concentrations (1 μM, 10 μM, or 50 µM) for 24 h. Equal volume of DMSO was used for experiment control. At the time of harvest on ice, all cells were washed three times with ice cold PBS following established protocols (Gyenis et al., 2011). One of the two DMSO controls (λ-phosphatase treated lysates) was harvested with the same lysis buffer but without any phosphatase inhibitors. Protein concentration was measured by BCA Protein Assay Kit (Pierce) using BSA as standard.

### SILAC Cell Incorporation

SILAC-dropout DMEM (Wisent) lacking l-arginine and l-lysine was supplemented with isotope-encoded l-arginine (Arg-6) (13C6) and l-lysine (Lys-4) (4.4,5,5-D4) (Silantes) at respective concentrations of 86.2 mg/L (0.398 mM) and 61.16 mg/L (0.274 mM) to create a “Medium” labelled cell medium (M). “Heavy” labelled medium (H) was created by supplementing the SILAC-dropout DMEM with isotope encoded l-arginine (Arg-10) (13C6, 15N4) and l-lysine (Lys-8) (13C6, 15N2) at respective concentrations of 87.7 mg/L (0.397 mM) and 52.4 mg/L (0.274 mM). “Light” labelled medium (L) was created using the SILAC-dropout DMEM and supplementing it with unlabeled l-arginine (R0) (83.9 mg/ml) and l-lysine (K0) (60.04 mg/ml). All media used for SILAC studies were supplemented with 10% of 10 kDa dialyzed FBS (Wisent), penicillin (100 U/mL), streptomycin (100 mg/ml) and l-proline (400 mg/ml, Cambridge Isotope Laboratories Inc.) to prevent arginine to proline conversion (Bendall et al., 2008). Cell media was filter-sterilized using 0.2 µm filter (Nalgene) prior to use for cell culture.

FT-U2OS cells expressing wild type CSNK2A1-HA were either adapted to “Light” (L) or “Medium” (M) SILAC media for 21 days and the isotope incorporation was verified by LC-MS/MS to be >95%. FT-U2OS cells expressing the inhibitor-resistant triple mutant form of CSNK2A1-HA was adapted to “Heavy” (H) SILAC media for 21 days and the isotope incorporation was verified by LC-MS/MS to be >95%. The WT (L, M) and TM (H) cell lines were treated with 1 μg/ml tetracycline for 48 h (cells were 80% confluent) to induce expression of the appropriate form of CSNK2A1. “Medium” WT and “Heavy” TM cells were treated with 30 µM CX-4945 for 4 h. “Light” WT cells were treated with equal volume of DMSO vehicle. Experiments were carried out with five biological replicates of each treatment.

### Phosphoproteome and Proteome Sample Preparation

Following treatment +/-CX-4945, the cells were lysed following the protocol established by Humphrey et al. (2015). Briefly, the cell media was vacuum-aspirated and cells were washed with ice cold PBS twice followed by lysis on ice in 1 ml of buffer containing 6 M guanidinium chloride (GdmCl), 100 mM tris pH 8.5, 10 mM tris (2-carboxyethyl)phosphine (TCEP), and 40 mM 2-chloroacetamide (CAA). Lysates were then heated for 5 min at 95°C, cooled on ice for 15 min, sonicated for 2 × 15 s, and heated again at 95°C for 5 min. Lysates were cleared for 10 min at 13,000 x *g* at 4°C. Lysate protein concentration was determined using a NanoDrop™ (Thermo).

An equal amount (0.5 mg) of lysate from each treatment—L, M, and H—were mixed for all five biological replicates. Proteins were then precipitated by chloroform (Sigma-Aldrich) and methanol (Fisher) extraction method (Wessel and Flügge, 1984) and protein pellets were resuspended in 1 ml of digestion buffer (5% 2,2,2-trifluoroethanol (TFE) (Aldrich) and 50 mM ammonium bicarbonate (FLUKA). Trypsin/Lys-C (Promega) was added at 1:100 enzyme:protein ratio and incubated for 4 h at 37°C with gentle agitation. Subsequently, additional trypsin (Promega) was added at 1:100 enzyme:protein ratio and the sample was incubated at 37°C for 16 h with gentle agitation. For phosphoproteome analysis, phosphopeptide enrichment was performed using titanium dioxide (TiO_2_) beads (Titansphere TiO Bulk 10 μm, Canadian Life Science) and styrenedivinylbenzene–reversed phase sulfonated (SDB-RPS) (3M Empore) StageTips, as described by Humphrey et al. (2015). Enriched phosphopeptide solutions were acidified using 0.1% formic acid and peptide concentrations were determined using a NanoDrop™ (Thermo). Lastly, 1 µg was injected for LC-MS/MS analysis. For proteome analysis, digested proteins were desalted using a C18 StageTip (3M Empore) (Rappsilber et al., 2007) and the peptide concentrations were determined using a NanoDrop™. Finally, 0.7 µg was injected for LC-MS/MS analysis.

### Mass Spectrometry

Phosphoproteome and proteome samples were analyzed using an Orbitrap Elite Hybrid Ion Trap-Orbitrap mass spectrometer (Thermo Scientific) connected to a NanoFlex (Thermo Electron Corp., Waltham, MA) nanospray ionization source with a source voltage of 2.4 kV. Samples were injected using a NanoAcquity UPLC (Waters) onto a Symmetry C18 trapping column (20 mm × 180 μm i. d., 5 μm, 100 Å) at a flow rate of 10 μL/min in 99% mobile phase A (0.1% FA (v/v), 1% mobile phase B (0.1% FA (v/v) in ACN) for 4 min. Samples were then separated on a NanoAcquity Peptide BEH C18 analytical column (250 mm × 75 μm i. d., 1.7 μm, 130 Å) at a flow rate of 300 nL/min by a linear gradient of increasing mobile phase B from initial condition 5%–7.5% over 1 min, followed by 7.5%–25% over 179 min, 25%–32.5% over 40 min, and culminating at 60% at 240 min. Mass spectrometer was operated by Thermo XCalibur software (version 2.7.0) in data-dependent acquisition (DDA) mode using an FT/IT/CID Top 20 scheme. MS1 survey scans were performed at 120,000 resolution from 400–1,450 m/z with full Automatic Gain Control (AGC) set to 10^6^ and an isolation window of 1.0 m/z. The top 20 most abundant ions were selected for MS2. A lock mass ion was enabled at 445.120025 m/z. Peptide ions were fragmented using a CID (Collision-Induced Dissociation) with Normalized Collision Energy (NCE) set to 35%, an activation time of 10 ms, and a Q value of 0.25. Data-dependent MS2 scans were acquired in the linear ion trap using rapid scan type, with a dynamic exclusion window of 30 s. Five biological replicates were analyzed without technical replicates.

### Data Analysis

Raw mass spectrometric data of the five biological replicates were analyzed using MaxQuant (version 1.6.2.10., Max Planck Institute of Biochemistry, Munich (Cox et al., 2009; Tyanova et al., 2016a) and searched against the Human UniProtKB database (9 May 2020), which contained 20,362 entries. Default settings of MaxQuant (Tyanova et al., 2016a) were used with minor changes: multiplicity was set to 3, medium labels were selected as Arg6 and Lys4 and heavy labels were selected as Arg8 and Lys10; maximum number of 3 missed cleavages was chosen for trypsin/P; carbamidomethyl (C) was set as a fixed modification while oxidation (M), acetylation (protein N-terminal), deamidation (NQ), and phosphorylation (STY) were set as variable modifications for phosphopeptide enriched samples while the proteome data were searched using analogue modifications yet excluding the phosphorylation (STY). For quantification, unmodified peptides as well as those with fixed and variable modifications, excluding phosphorylation (STY), were included. “Re-quantify” and “Match Between Runs” were selected using default parameters. The maximum number of variable modifications was set to 5. The minimum score for modified peptides was set to 40, and the decoy-database setting was “Revert.” The False Discovery Rate (FDR) of PSM, protein and site of 0.01 was used.

Output data from MaxQuant were interpreted using Perseus software (version 1.6.14.0) (Max Planck Institute of Biochemistry, Munich) as described (Tyanova et al., 2016b) (https://maxquant.net/perseus/). Briefly, the light condition (“L”) comprised CSNK2A1-WT subjected to DMSO treatment, the medium condition (“M”) comprised CSNK2A1-WT subjected to CX-4945 treatment, and the heavy condition (“H”) comprised CSNK2A1-TM subjected to CX-4945 treatment. The Phospho (STY)Sites.txt table was annotated with PTMSigDB (Krug et al., 2019). Normalized ratios of medium to light (M/L), heavy to light (H/L), and heavy to medium (H/M) intensities from the Phospho (STY)Sites.txt output were log2 transformed. Phosphorylation sites were considered for further analysis only if the localization probability was ≥0.75. Reverse sequences and contaminants were removed. Our differential expression filtering workflow was the following: Phosphopeptides were considered significantly downregulated and significantly upregulated if mean M/L < 0 and mean M/L > 0, respectively (One-sample *t*-test of M/L values, Benjamini–Hochberg (BH) FDR multiple testing correction q < 0.05). Phosphopeptides were inhibited or upregulated to a biologically relevant level if the M/L log2 value < -0.585 or M/L log2 value >0.585 (1.5-fold regulation), respectively.

Phosphopeptides were considered ‘rescued’ if -0.585 < mean H/L < 0.585 (One-sample *t*-test of H/M values, q < 0.05 BH FDR). Several phosphopeptides were significantly downregulated 1.5-fold but did not satisfy our strict biological criterion for ‘rescue’. Instead, they demonstrated ‘partial rescue’ (H/M > 0) (One-sample *t*-test H/M, q < 0.05 BH FDR). Therefore, partially rescued phosphopeptides include those which were significantly upregulated in cells expressing exogenous CSNK2A1-TM in the presence of CX-4945 (H), but not to the same level as cells overexpressing exogenous CSNK2A1-WT in the absence of any inhibitor (L).

Proteins inhibited and upregulated—and subsequently rescued—were determined using the same criteria, except applied to the normalized log2 transformed M/L, H/L, and H/M ratios originating from the proteinGroups.txt MaxQuant output.

Further interpretation and visualization of our filtered phosphoproteomic and proteomic data were conducted using R Studio (Version 1.2.5033; RStudio Team (2020). RStudio: Integrated Development for R. RStudio, PBC, Boston, MA URL http://www.rstudio.com/). Sequence logo analysis of phosphopeptides was conducted using the WebLogo tool (University of California, Berkeley, https://weblogo.berkeley.edu/logo.cgi) (Crooks et al., 2004). Network creation was performed using the online GeneMANIA tool (University of Toronto, https://genemania.org/) (Warde-Farley et al., 2010). An “equal by data type” weighting method was utilized, and the following networks were utilized for network creation: physical interaction, genetic interaction, shared protein domains, co-localization, pathway, and predicted, as well as our own dataset. Protein substrates which were identified by our rescued phosphopeptides were assigned a value of 1 to indicate an interaction. If a partially rescued phosphopeptide could have originated from several proteins (e.g., CDK1, CDK2, or CDK3), then all proteins were included in the uploaded gene list at a value of 1. Gene Ontology (GO) terms, which were enriched among the proteins depicted in the GeneMANIA network (protein substrates of CSNK2 and those implicated in relevant cellular signalling), were visualized using R Studio.

## Results

### Comparison of CSNK2 Inhibitors by Western Blotting

**FIGURE 1:**
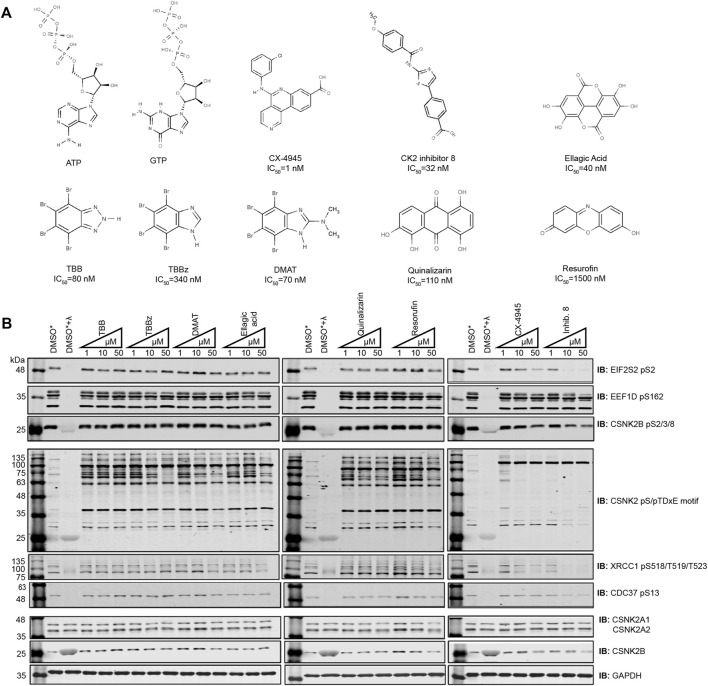
Comparing the efficacy of CSNK2 inhibitors in U2OS cells. **(A)** Schematic illustration of the CSNK2 inhibitors that were evaluated in this study with reported *in vitro* IC_50_ values **(B)** CSNK2 inhibition was assessed by immunoblotting with the indicated phospho-specific antibodies after 24 h of inhibitor CX-4945 treatment at the indicated concentrations in U2OS cells. Lysis buffer of lysates marked with * did not contain phosphatase inhibitors. DMSO treated lysates +/-λ-phosphatase were used as immunoblotting controls for the phospho-specific antibodies. Results are representative of two independent experiments.

To compare the potency of CSNK2 inhibitors, cells were treated with eight commercially available and previously published compounds (TBB, TBBz, DMAT, Ellagic Acid, Quinalizarin, Resorufin, Inhibitor 8, CX-4945) and lysates were analyzed by immunoblotting (Figure 1A). We treated human osteosarcoma (U2OS) cells with 1 μM, 10 μM, and 50 μM (Figure 1B) and HeLaT cells with 1 μM, 10 μM, and 20 μM (Supplementary Figure S1) of each inhibitor for 24 h. We subsequently compared the level of CSNK2 inhibition using a panel of commercial phospho-specific antibodies (CSNK2 pS/pTDXE motif, XRCC1 pS518/T519/T523, CDC37 pS13) as well as antibodies we developed for sites previously validated to be CSNK2-dependent (EIF2S2 pS2 (Gandin et al., 2016), EEF1D pS162 (Gyenis et al., 2011)) (Figure 1B, Supplementary Figure S1, S2A). Additionally, we developed a phosphospecific antibody for CSNK2B pS2/3/4/8 to monitor CSNK2A1-dependent auto-phosphorylation of the regulatory beta subunit (Figure 1B, Supplementary Figure S1, S2B). We immediately noticed disparities in potency of cellular CSNK2 inhibition. Six of eight CSNK2 inhibitors demonstrated little to no inhibition of CSNK2 at the tested concentrations in U2OS cells as indicated by our immunoblotting results (Figure 1B and Supplementary Figure S1). By comparison, Inhibitor 8 and CX-4945 showed dose-dependent inhibition of CSNK2. Inhibitor 8 demonstrated the strongest inhibition of CSNK2 at all three tested concentrations in these evaluations (Figure 1B and Supplementary Figure S1). Similar results were seen in human adenocarcinoma HeLaT cells (Supplementary Figure S1), confirming that our results were not restricted to U2OS cells.

Despite effective inhibition of CSNK2 by CX-4945 and Inhibitor 8, the phosphoantibody recognizing EEF1D pS162 did not demonstrate reduced phosphorylation. In our previous study in which we demonstrated EEF1D pS162 to be a *bona fide* substrate of CSNK2 in cells, *in vitro* phosphorylation by CSNK2 at this site was dramatically increased following λ-phosphatase treatment of EEF1D immunoprecipitates. This observation demonstrates high occupancy of the site in living cells suggesting that the site is not readily acted upon by phosphatases (Gyenis et al., 2011).

Although we observed Inhibitor 8 to be the most potent inhibitor of CSNK2 in these experiments, we chose CX-4945 for use in our systematic CSNK2 substrate identification and validation approach for a number of reasons. CX-4945 was previously demonstrated to have a favorable specificity profile *in vitro*. When tested against a panel of 238 kinases, only 7 were inhibited greater than 90% at a concentration of 500 nmol/L. CSNK2A1 and CSNK2A2 exhibited the lowest IC_50_ of the 7 kinases inhibited at 1 nmol/L CX-4945 (Siddiqui-Jain et al., 2010). Furthermore, CX-4945 is the only small molecule CSNK2 inhibitor of those we tested which is currently in clinical trials (NCT04663737, NCT04668209, NCT03904862). CSNK2-independent cellular effects of CX-4945 have been well documented (Kim et al., 2014; Kim et al., 2016; Lertsuwan et al., 2018), and while they may in part be responsible for clinical efficacy of the inhibitor, they are detrimental to definitive kinase-substrate assignment when employed without a robust method of validation. When utilized with CX-4945, our strategy might therefore provide some insight on the extent to which CSNK2-dependent inhibition contributes to the clinical efficacy of this inhibitor. Taken together, we reasoned CX-4945 was a suitable CSNK2 inhibitor for the development of a systematic CSNK2-substrate identification and validation workflow.

### Employing Chemical Genetics for CSNK2 Kinase Substrate Validation

**FIGURE 2:**
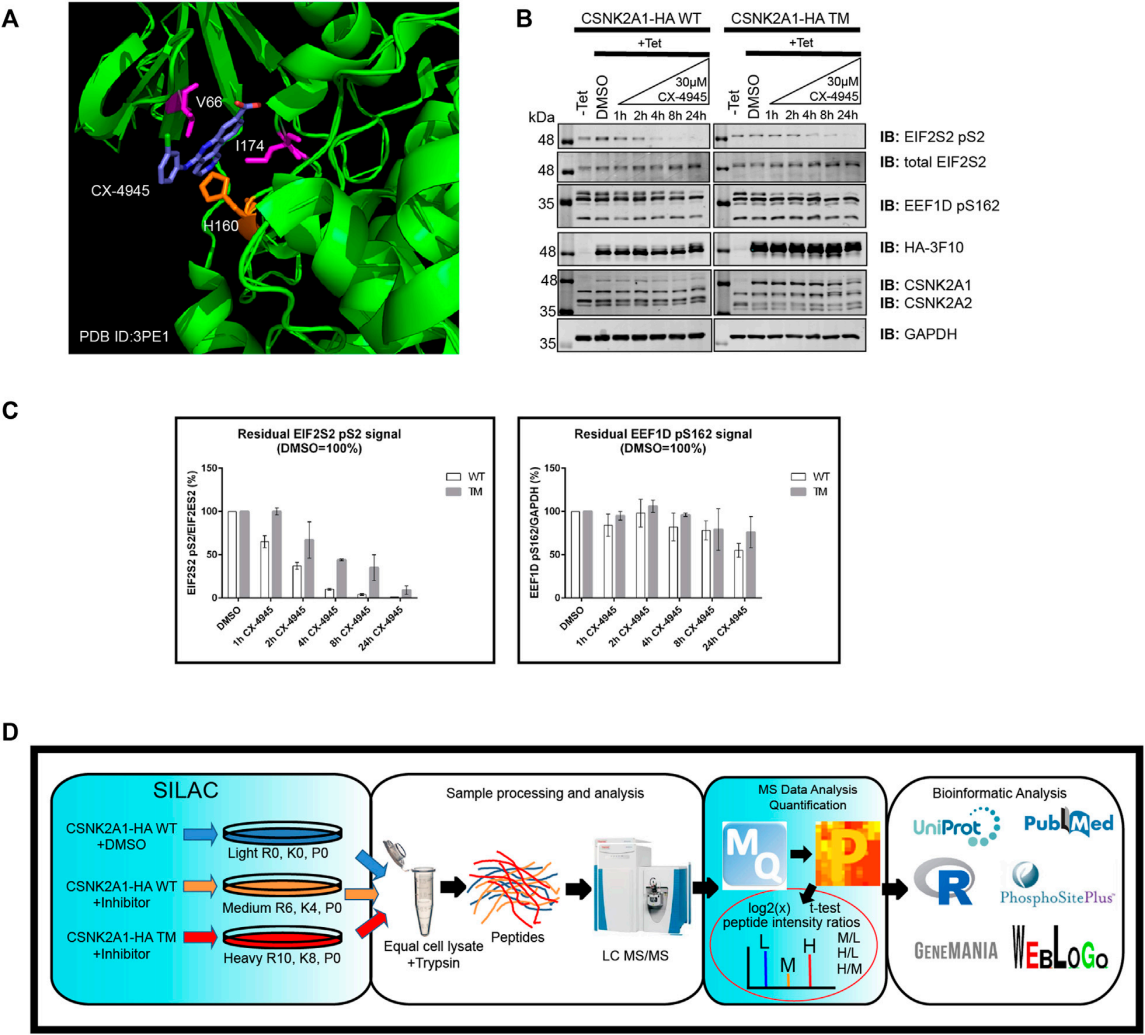
Use of a triple mutant CSNK2A1 for validation of CSNK2 substrates. **(A)** Crystal structure of CX-4945 interacting with the ATP-binding site of CSNK2A1 (PDB ID: 3PE1). Highlighted are the residues mutated in CSNK2A1-TM (V66A/H160D/I174A) to create an inhibitor tolerant/resistant kinase. **(B)** Immunoblots demonstrate the inhibition and rescue of CSNK2-dependent phosphorylation using Flp-In T-REx U2OS cell lines stably expressing CSNK2A-HA WT or CSNK2A-HA TM with tight Tetracycline (Tet-ON) regulation. Cells were treated with 30 µM of CX-4945 for the indicated times. **(C)** Bar charts demonstrate residual CSNK2 activity based on EIF2S2 pS2/total EIF2S2 or EEF1D pS162/GAPDH ratios, with CSNK2 activity in the DMSO control defined as 100%. Each column represents the mean value of two independent experiments with range bars displayed. Band intensities on blots were quantified with LiCor Odyssey v3.0 software. Results are representative of two independent experiments **(D)** Overview of large-scale identification and validation of CSNK2 substrates using a chemical genetics approach combined with triple SILAC quantitative phosphoproteomics.

To start, we developed Flp-In T-REx U2OS (FT-U2OS) cell lines expressing the CSNK2A1-HA wild-type (CSNK2A1-WT) or CSNK2A1-HA triple mutant (CSNK2A1-TM, V66A/H160D/I174A) form of the kinase with tight tetracycline regulation (Figures 2A,B; Supplementary Figure S4). When characterizing these cell lines for their response to CX-4945 treatment, we observed the earliest inhibition of CSNK2A1-WT occurred at 4 h post-treatment with 30 µM CX-4945 using the phospho-EIF2S2 pS2 antibody (Figure 2B). This inhibitory effect on CSNK2 was then ‘rescued’ in the CSNK2A1-TM cell line under the same conditions (Figures 2B,C). Therefore, we proceeded to treat cells with 30 µM of CX-4945 for 4 h in our phosphoproteomics evaluation (outlined in Figure 2D).

To identify phosphopeptides which were diminished following CX-4945 treatment, normalized intensity ratio values were calculated between CSNK2A1-WT cells treated with CX-4945 and CSNK2A1-WT cells treated with DMSO (control). To validate phosphopeptides as CSNK2-dependent, normalized intensity ratio values were calculated between CSNKA1-TM cells treated with CX-4945 and CSNK2A1-WT cells treated with DMSO (control). Those phosphopeptides which maintained phosphorylation in cells expressing CSNKA1-TM despite the presence of CX-4945 were designated as rescued. Using this method, we could identify and validate *bona fide* CSNK2 substrates.

### Identification and Validation of CSNK2 Substrates

**FIGURE 3:**
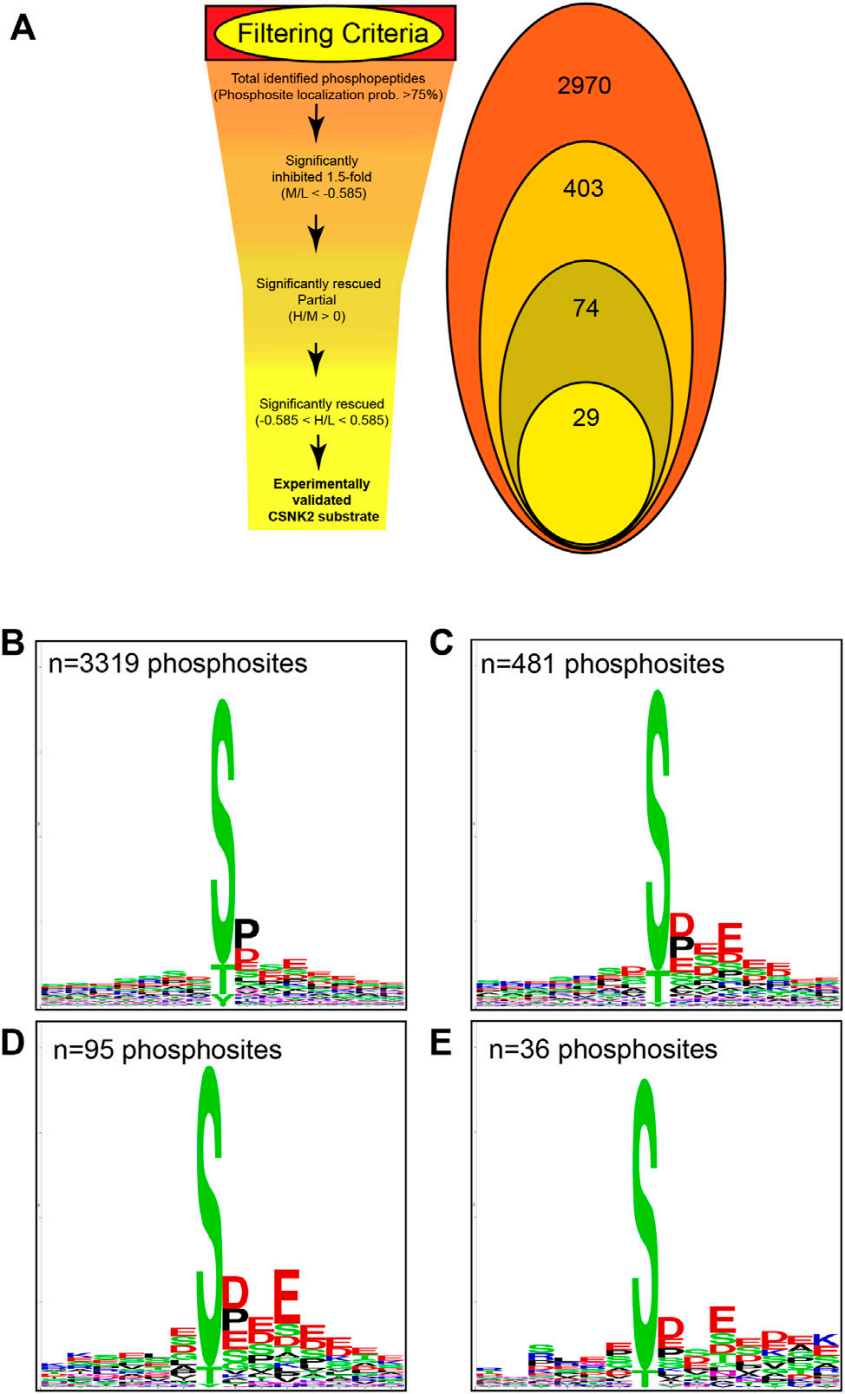
Filtering criteria utilized in CSNK2 substrate identification and validation. **(A)** Filtering criteria utilized to identify CSNK2-dependent phosphopeptides. The number of distinct phosphopeptides that remained after each filtering step is displayed in the Venn diagram variation. WebLogo consensus sequence analysis was conducted on phosphosites identified **(B)** prior to filtering, **(C)** after filtering for phosphopeptides significantly inhibited, **(D)** after filtering for phosphopeptides demonstrating partial rescue, and **(E)** after filtering for phosphopeptides demonstrating rescue. The n values represent the number of phosphosites used for consensus sequence analysis.

**FIGURE 4:**
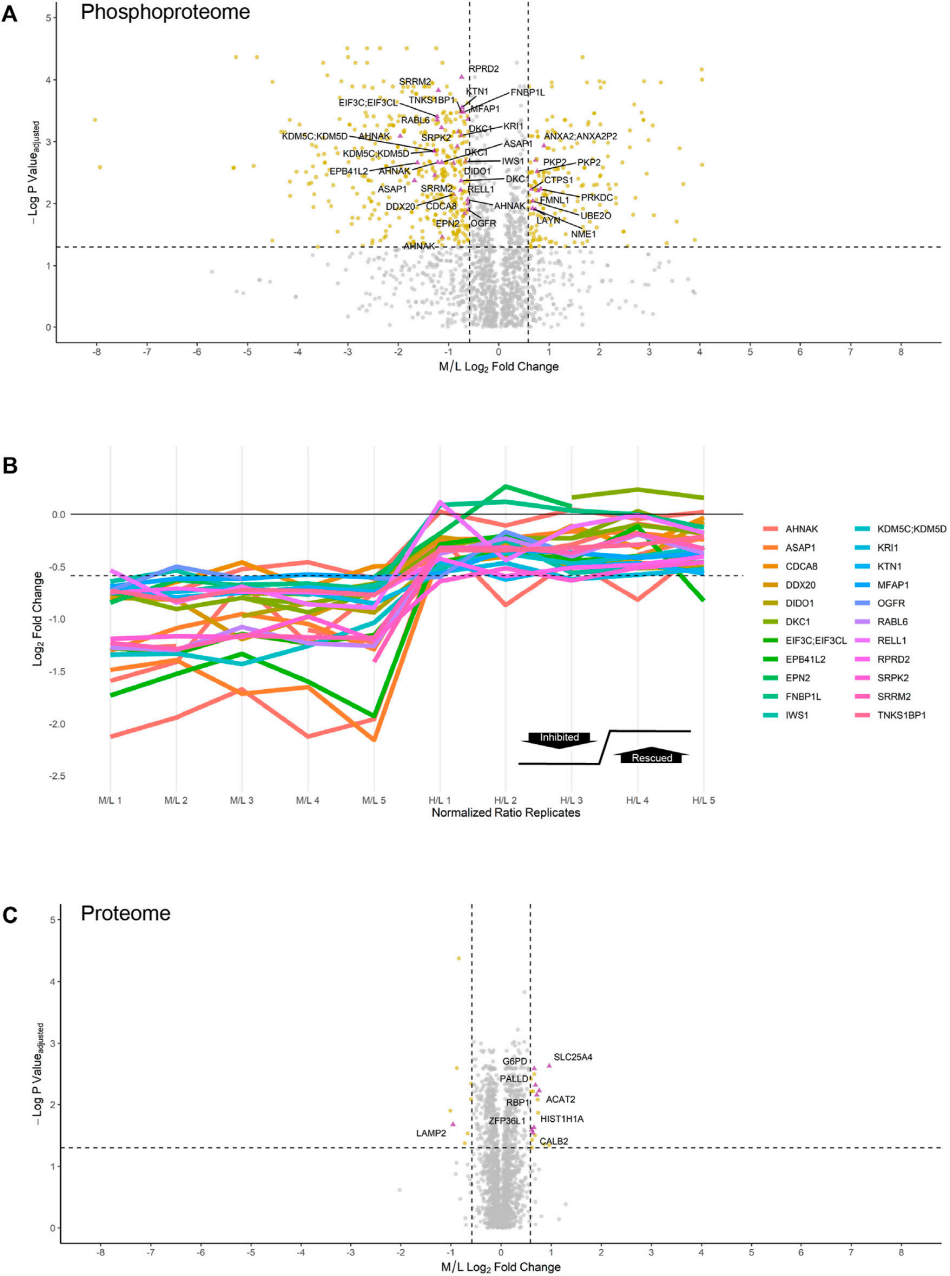
Inhibition and rescue profile of CSNK2 substrates. Differential expression of **(A)** phosphopeptides and **(C)** proteins after treating U2OS cells expressing CSNK2A1-WT with 30uM of CX-4945 for 4 h. Phosphopeptides/proteins highlighted in gold are significantly down- or upregulated 1.5-fold. Phosphopeptides/proteins highlighted in purple demonstrate rescue in U2OS cells expressing CSNK2A1-TM with 30uM of CX-4945 for 4 h. Rescued phosphopeptides are labelled with the gene name of the corresponding protein. Vertical dashed lines (−/+ 0.585) represent 1.5-fold down- and upregulation, respectively. The horizontal dashed line represents an adjusted *p*-value cutoff of 0.05 **(B)** Inhibition and rescue profiles of CSNK2 substrate phosphopeptides. The horizontal dashed line is representative of significant inhibition (-0.585).

Using the MaxQuant-Andromeda integrated computation platform, we identified 4,511 proteins and 3,319 distinct phosphosites corresponding to 2,970 distinct phosphopeptides. We performed downstream bioinformatic analyses of the MaxQuant output using Perseus (Figure 3A). Data tables were annotated with PTMSigDB, a database which contains curated, site-specific signature data regarding perturbation, kinase activity, and signalling pathways. Consensus sequence analysis of distinct phosphosites was performed at each filtering step using WebLogo (Figure 3B) (Crooks et al., 2004). Following initial removal of reverse sequences and contaminants, log2 transformation, and localization probability filtering for >75%, 403 distinct phosphopeptides (481 distinct phosphosites) were significantly downregulated by 1.5-fold or greater (M/L < -0.585) following 4 h of 30 µM CX-4945 treatment (One-sample *t*-test M/L, q < 0.05 BH FDR). Of these 403 phosphopeptides significantly downregulated 1.5-fold or greater, 29 distinct phosphopeptides mapping to 22 proteins (36 distinct phosphosites) demonstrated rescue according to our biological criterion (-0.585 < H/L < 0.585) (One-sample *t*-test H/M, q < 0.05 BH FDR) (Figure 4A; Supplementary Figure S5). Of the 36 distinct phosphosites identified, 30 phosphosites (∼83%) adhered to the minimal CSNK2 recognition motif further reinforcing the promise of this strategy. Rescued phosphopeptides were cross referenced with proteomic analysis of the same samples to ensure that differential abundance of these phosphopeptides was not a result of differential protein expression (Supplementary Figure S6).

Of note, 177 distinct phosphopeptides (200 distinct phosphosites) were significantly upregulated 1.5-fold or greater (M/L > 0.585) following 30 µM CX-4945 treatment (One-sample *t*-test M/L, q < 0.05 BH FDR). Of these 177 phosphopeptides significantly upregulated 1.5-fold or greater, nine distinct phosphopeptides (10 distinct phosphosites) demonstrated rescue (-0.585 < H/L < 0.585) (One-sample *t*-test H/M, q < 0.05 BH FDR) (Figure 4A, Supplementary Table S1).

Altogether, we were able to identify and validate 22 CSNK2 substrates which correspond to 29 phosphopeptides having demonstrated robust inhibition and rescue (Figure 4B). Out of 29 phosphopeptides which were identified to be CSNK2-dependent, 11 phosphopeptides were of multiplicity 1 (i.e., one phosphorylation site present on the identified peptide). Thus, our method identified 11 high-confidence CSNK2 phosphosite substrates within a subset of the proteins identified to be CSNK2-substrates.

Several phosphopeptides were significantly downregulated 1.5-fold but did not satisfy our strict biological criterion (-0.585 < H/L < 0.585). Instead, they demonstrated ‘partial rescue’ (H/M > 0) (One-sample *t*-test H/M, q < 0.05 BH FDR). Partially rescued phosphopeptides include those which were significantly upregulated in cells expressing exogenous CSNK2A1-TM in the presence of CX-4945, but not to the same level as cells overexpressing exogenous CSNK2A1-WT in the absence of any inhibitor. Considering this filtering was less stringent than that which was applied to our list of rescued phosphopeptides, this list of partially rescued substrates naturally also included those which were rescued (-0.585 < H/L < 0.585). In total, our partially rescued list of substrates identified 55 protein substrates, 74 distinct phosphopeptides, and 95 distinct phosphosites. This list warrants further attention and serves as a potential source of *bona fide* CSNK2 substrates (Supplementary Table S1).

### Motif Analysis of CSNK2-dependent Phosphosites

Kinase assays performed with purified components *in vitro* have repeatedly demonstrated CSNK2 activity to highly favor the presence of acidic residues downstream of the phosphoacceptor. One notable negative sequence determinant of CSNK2-catalyzed phosphorylation has been reported to be the presence of Proline at the +1 position. We conducted consensus sequence analysis using the online WebLogo tool at each filtering step to visualize the phosphorylation landscape of cellular CSNK2 substrates (Crooks et al., 2004). The sequence generated from our list of rescued phosphopeptides closely adheres to the minimal consensus sequence of CSNK2 frequently noted in the literature—[S/T]-x-x-[D/E] (Figure 3B) (Meggio and Pinna, 2003; Salvi et al., 2009). The high frequency of aspartic acid and glutamic acid residues downstream from the phosphoacceptor residue reflects the well-characterized acidophilic nature of CSNK2. Upstream residues do not seem to demonstrate a high level of conservation and are likely less important to CSNK2 phosphorylation, as also previously noted in the literature. Although CSNK2 has demonstrated the ability to phosphorylate tyrosine residues in previous studies (Vilk et al., 2008), we did not see phosphorylated tyrosine sites following our stringent filtering.

Despite strong adherence of identified phosphosites to the typical CSNK2 recognition motif, our WebLogo analysis demonstrated that proline is quite prevalent at position +1 despite previous studies claiming it is a negative determinant (Marin et al., 1992). While we do not suggest that proline at this position positively influences phosphorylation, it is possible that proline at the +1 position is not enough of a negative sequence determinant to absolutely prevent CSNK2 phosphorylation *in vivo*.

### Characterizing the CSNK2-dependent Proteome

In comparison to the phosphoproteome, the proteome was minimally perturbed in response to CX-4945 treatment (Figures 4A,C). Only a small number of proteins were differentially regulated in a CSNK2-dependent manner. Of the 4,511 proteins identified and quantified by MaxQuant, only eight proteins were significantly downregulated 1.5-fold or greater (M/L < -0.585) (One-sample *t*-test M/L, q < 0.05 BH FDR). One of these proteins, lysozome-associated membrane glycoprotein 2 (LAMP2), demonstrated rescue (-0.585 < H/L < 0.585) (One-sample *t*-test H/M, q < 0.05 BH FDR). In contrast, proteins which were significantly upregulated 1.5-fold or greater (M/L > 0.585) in response to CX-4945 treatment numbered 19 (One-sample *t*-test M/L, q < 0.05 BH FDR). Eight of these proteins demonstrated rescue (-0.585 < H/L < 0.585) (One-sample *t*-test H/M, q < 0.05 BH FDR) (Figure 4B, Supplementary Table S2).

### Development of Novel Antibodies for Bona Fide CSNK2 Substrates

**FIGURE 6:**
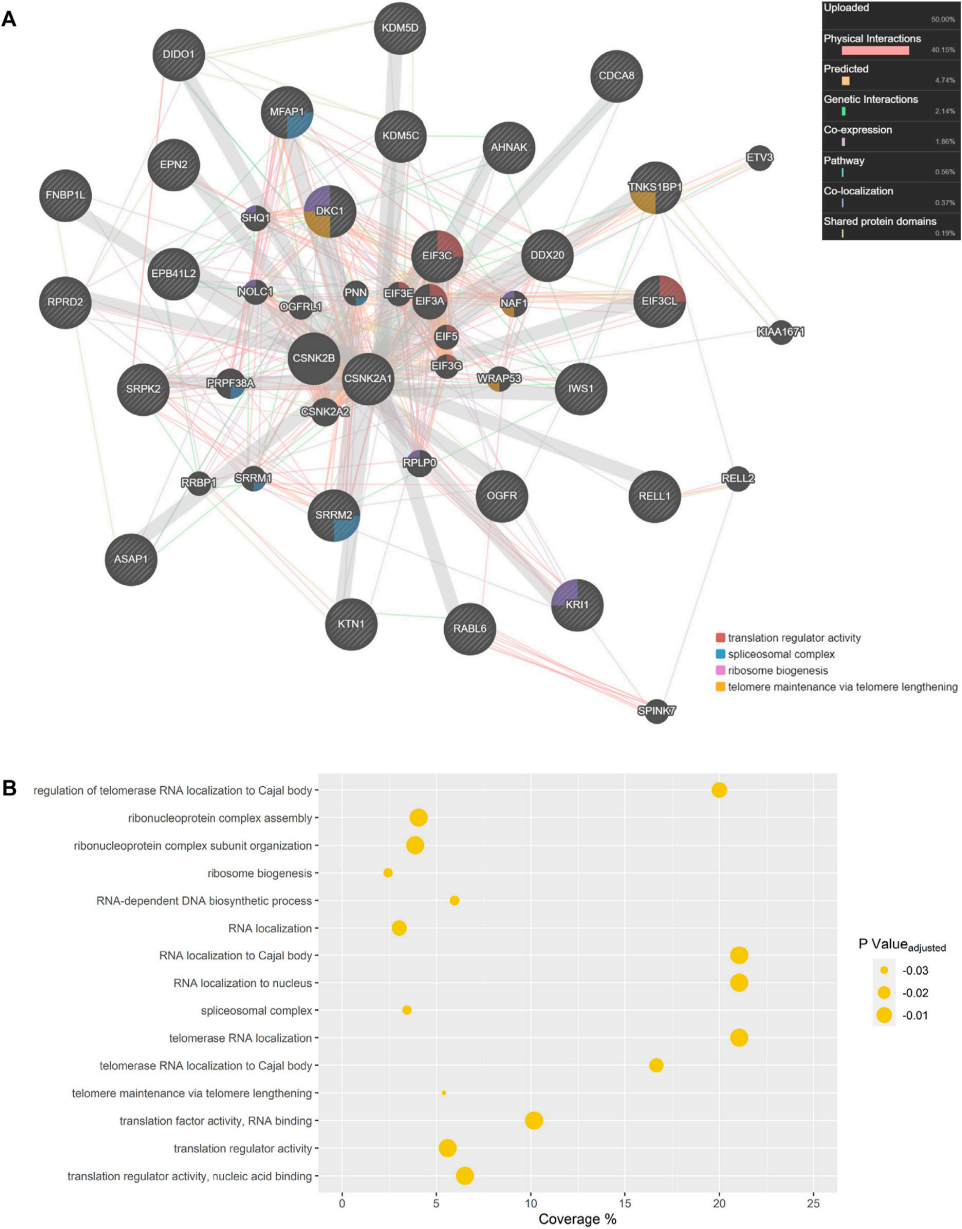
CSNK2A1 Interaction Network. **(A)** Rescued CSNK2 protein substrates and related proteins are depicted in a GeneMANIA network. Previous database information regarding physical interactions (red), co-expression (purple), predicted interactions (orange), genetic interactions (green), co-localization (blue), and shared protein domains (yellow) were employed in network creation in an “equal by data type” weighting approach. Our own list of rescued substrates was utilized in network creation and each assigned a value of 1. If a phosphopeptide was not unique and could have originated from several proteins, all potential proteins were assigned a value of 1. Hashed gene names indicate proteins included in our query list, while those with a solid background are related proteins inserted during GeneMANIA network creation. Proteins implicated in Gene Ontology (GO) biological processes are labelled. **(B)** GO terms enriched amongst proteins displayed in the GeneMANIA network. Coverage represents the proportion of displayed proteins which are annotated with a certain GO term when compared to all genes in the genome annotated with that same term.

To extend this work, we developed novel antibodies against a number of putative CSNK2 substrates identified by the chemical genetics strategy that we devised. When deciding on antibody candidates from those identified and validated within our MS analysis, our selection criteria also included: i) (phospho)peptide uniqueness, ii) molecular weight of the protein, iii) reported cellular abundance of the protein, iv) adherence to CSNK2 motif, and v) literature relating the site to CSNK2. We also utilized our list of partially rescued sites as a source of candidates. We developed and characterized phospho-specific antibodies for DNA ligase 1 (LIG1) pS66 (ARVLGpSEGEEE) and Lupus La protein (SSB) pS366 (KTKFApSDDEHD) (Supplementary Figure S3A). Each of these phosphoproteins exhibited significant inhibition and subsequent ‘rescue’ following 4 hours of CX-4945 treatment in the CSNK2A1-WT and CSNK2A1-TM cell lines, respectively (Figure 6). These candidates originated from the list of partially rescued sites identified by our MS analysis, thus further increasing our confidence in the *bona fide* nature of this list of substrates. During the course of phospho-specific antibody development, we also obtained an anti-SSB antibody which selectively recognizes the unphosphorylated version of the peptide (KTKFASDDEHD) (Figure 6 and Supplementary Figure S3B). This antibody demonstrated a decrease in phosphorylation at S366 with increased CX-4945 incubation times, perfectly opposing the trend seen with the antibody targeting SSB pS366. With these newly developed phospho-specific antibodies, we gained additional tools to monitor CSNK2 activity and further increased confidence in our kinase-substrate identification method.

### CSNK2A1 Interaction Network

Biological network representation can simultaneously display several interaction types which occur between constituents in living cells by extracting data from numerous databases and existing literature. Network depictions can also provide meaningful information and leads on potential CSNK2 interactors and downstream effector molecules. Therefore, using GeneMANIA, we generated a CSNK2A1 interaction network of the proteins which were rescued in our analysis (Figure 6A) (Warde-Farley et al., 2010). Previous database information regarding physical interactions, co-expression, genetic interactions, co-localization, and shared protein domains, as well as predicted interactions, were employed in network creation with an “equal by data type” weighting. Our own uploaded dataset was also utilized in network creation; rescued CSNK2A1 protein substrates were assigned a value of 1. If a phosphopeptide was not unique and could have originated from several proteins (e.g., EIF3C or EIF3CL) all potential proteins were included in the uploaded gene list at a value of 1. The network displays 26 proteins which were manually inputted and 19 proteins which were implicated by some relationship to our proteins of interest. Proteins displayed in this network were enriched for several GO terms, including numerous biological processes with which CSNK2 has previously been associated (Figure 6B).

## Discussion

Collective efforts to characterize the CSNK2-dependent phosphoproteome have resulted in no shortage of phosphoproteomic studies directed at identifying putative substrates using CSNK2-directed inhibitors. These inhibitors exhibit variable specificity, display off-target effects, and they are typically not accompanied by rigorous validation. In this study, we describe a strategy for the systematic identification and validation of CSNK2 substrates by employing an inhibitor-resistant mutant of CSNK2 mutant in conjunction with triple SILAC phosphoproteomics.

We began by evaluating the ability of several commercially available CSNK2 inhibitors to effectively inhibit CSNK2 in U2OS and HeLaT cells. Using multiple phospho-specific antibodies recognizing previously determined *bona fide* CSNK2 substrates, we demonstrated that only two of eight CSNK2 inhibitors—CX-4945 and Inhibitor-8—were effective in inhibiting CSNK2 in cells. These observations fail to demonstrate utility of a number of these compounds for cell-based studies despite previous *in vitro* studies demonstrating strong potency of these compounds as inhibitors of CSNK2 in enzymatic assays (Sarno et al., 2001; Pagano et al., 2004a; Pagano et al., 2004b; Cozza et al., 2006; Cozza et al., 2009; Sandholt et al., 2009). The potency of small molecule inhibitors in living cells can be vastly different from their *in vitro* inhibition profiles, thereby highlighting the importance of testing inhibitors in cell-based assays using validated CSNK2 activity markers. With the increased identification of *bona fide* CSNK2 substrates in cells, our repertoire of CSNK2 activity markers will also expand to serve this purpose.

Evaluating CX-4945 with the chemical genetics validation strategy described here, we identified 29 phosphopeptides which were both inhibited in cells expressing CSNK2A1-WT in the presence of CX-4945 and also rescued (i.e., maintained) in cells expressing CSNK2A1-TM in the presence of CX-4945. This led to the identification of 22 CSNK2 protein substrates (Supplementary Figure S5). For some of these protein substrates, it is not exactly clear which protein isoform from which the phosphopeptide originates. For example, QPLLLpSEDEEDTKR is a multiplicity 1 CSNK2 phosphopeptide substrate which was inhibited and rescued, yet this tryptic peptide is present in both EIF3C and EIF3CL. Moreover, numerous tryptic phosphopeptides which demonstrated rescue were multiply phosphorylated (multiplicity >1), making confident identification of the CSNK2-dependent phosphosite(s) challenging. Despite these limitations, several phosphopeptides of multiplicity 1 were also rescued, and we were therefore able to definitively identify 11 CSNK2 phosphosite substrates. None of these sites have been previously validated to be CSNK2-dependent phosphosites, highlighting the utility of this strategy to identify and validate novel CSNK2 sites. While we acknowledge that some of the phosphopeptides which demonstrated inhibition and rescue may be downstream effects of CSNK2 inhibition (i.e., due to sequential phosphorylation by CSNK2-activated kinases), a high proportion (∼83%) of phosphosites identified adhere to the minimal CSNK2 recognition motif.

Several phosphopeptides were significantly downregulated 1.5-fold but did not satisfy our strict biological criterion for ‘rescue’ (-0.585 < H/L < 0.585). Instead, they demonstrated ‘partial rescue’ (H/M > 0) (One-sample *t*-test H/M, q < 0.05 BH FDR). We successfully raised antibodies against the following sites which were identified in our list of partially rescued substrates: ARVLGpSEGEEE (LIG1 pS36), KTKFApSDDEHD (SSB pS366), and KTKFASDDEHD (SSB S366). A dose-dependent decrease in both phosphoproteins was observed with increasing concentrations of CX-4945 (and in the case of SSB, a coordinated increase in the unphosphorylated protein variant) using these antibodies. These effects were then rescued in cells expressing CSNK2A1-TM in the presence of CX-4945, thus confirming that these phosphosites are *bona fide* CSNK2 sites. CSNK2 was previously demonstrated to phosphorylate the N-terminal region of LIG1 *in vitro* resulting in an increase in LIG1 activity (Prigent et al., 1992). LIG1 pS66 is part of a strong CSNK2 consensus sequence and was subsequently shown to be a CSNK2 site *in vitro* (Rossi et al., 1999). To our knowledge, our systematic method of CSNK2 substrate identification and validation is the first to confirm this specific site to be a true cellular substrate of CSNK2. CSNK2-dependent phosphorylation of SSB pS366 has been extensively characterized and previously validated *in vivo* (Fan et al., 1997; Schwartz et al., 2004). SSB plays a crucial role in the termination and re-initiation of the RNA polymerase 3 complex by binding the poly Uridine 3′ tails of nascent polypeptides. With the development and characterization of these novel phospho-specific antibodies, we demonstrated the partially rescued list of substrates to be a source of *bona fide* CSK2 substrates. Remarkably, 28 phosphopeptides of multiplicity 1 are present in this list of partially rescued phosphopeptides, providing site-specific information regarding CSNK2 activity in living cells (Supplementary Figure S7).

Other previously known CSNK2 substrates were also identified in our list of partially rescued phosphopeptides, increasing confidence in the *bona fide* nature of this list to the same effect. The pS109 site of ATP-binding cassette 1 (ABCF1/ABC50) was identified in our analysis and has been shown to be phosphorylated by CSNK2 *in vitro*. Phosphoablative mutation of this site to alanine resulted in a marked decrease in EIF2S2 binding to 80S ribosomes and polysomal fractions, identifying a role for CSNK2 in regulation of mRNA translation (Paytubi et al., 2008). Additionally, translation initiation factors EIF2S2 and EIF5B (also identified in our list of partially rescued substrates) have also been previously reported to be *bona fide* CSNK2 substrates (Wang et al., 2001; Majumdar et al., 2002; Llorens et al., 2003; Homma et al., 2005; Gandin et al., 2016). The pS121 and pS122 sites of protein phosphatase inhibitor 2 (PPP1R2) were also identified in our analysis, and they have too been previously determined to be CSNK2 sites using radiolabeled [γ -32P] ATP *in vitro* (Holmes et al., 1986).

To gain insight regarding the involvement of CSNK2 in cellular processes, we conducted network analysis on the list of rescued protein substrates (Figure 6A), and the list of partially rescued protein substrates (Supplementary Figure S8). The importance of these depictions is that they allow for integrative visualization of network constituents using multiple different types of interaction data including physical interactions, predicted interactions, genetic interactions, pathway information, co-localization data, and shared protein domains. Several GO terms were enriched among the proteins depicted in the GeneMANIA network of both rescued (Figure 6B; Supplementary Table S3) and partially rescued protein substrates (Supplementary Figure S9; Table S4) including translation regulator activity, spliceosomal complex, ribosome biogenesis, telomere maintenance via telomere lengthening, and several other biological processes which have been previously associated with CSNK2. An added benefit was the ability of GeneMANIA to highlight studies which demonstrated large overlap in physical CSNK2 interactors. Numerous phosphopeptides which demonstrated partial rescue (including a few that satisfied our strict criterion for rescue) were previously identified as CSNK2 substrates in a study by Zhang and colleagues (Zhang et al., 2011). In the study by Zhang et al., proteins from HeLa cell lysate were immobilized onto solid-phase beads and phosphorylated by a recombinant CSNK2 heterotetramer composed of catalytic alpha subunits. Tryptic phosphopeptide substrates were obtained following digestion and MS analysis. The identification of thirteen phosphosites (underscore followed by number indicates the multiplicities of their respective phosphopeptides) overlapped with those identified and validated in our partially rescued list. These include the following (*Site_Multiplicity*): DHX16 *S103_2*, EIF5B *S214_1*, EXOSC9 *S306_1*, IWS *S398_2* and IWS *S400_2*, MFAP *S52_2* and MFAP *S53_2*, NOP58 *S502_1*, PRCC *S157_2*, RPRD2 *S374_1*, SSB *S366_1*, and TNKS1BP1 *S1620_2* and TNKS1BP1 *S1621_2*. The following sites identified on partially rescued phosphopeptides in our dataset also overlapped but were identified on phosphopeptides of differing multiplicities in the Zhang study: DKC1 *S451_2* and DKC1 *S453_2*, NOP56 *S519_1*, and SRRM1 *S874_1*. Such extensive overlap with this *in vitro* lysate study further demonstrates that our list of partially rescued phosphosites is a source of CSNK2 cellular substrates.

In addition to identifying numerous CSNK2 substrates which are downregulated and rescued, nine phosphopeptides were upregulated in response to CX-4945 treatment with restoration of baseline phosphorylation levels in the presence of CSNK2A1-TM. Strikingly, eight of these nine peptides presented with a CSNK2 recognition motif. Cell systems have previously been demonstrated to enact compensatory mechanisms in response to kinase inhibition. This might include upregulating other kinases which directly compensate for reduced CSNK2 activity. It is also possible that the sequences which flank these phosphosites contain positive sequence determinants for other kinases, or that the spatial regulation of these proteins is altered in response to CSNK2 inhibition such that phosphorylation by a nearby kinase is favoured. CSNK2 has also been demonstrated to prime phosphorylation by other kinases at nearby residues—and vice versa—in what is known as hierarchical phosphorylation (St-Denis et al., 2015; Nuñez de Villavicencio-Diaz et al., 2017). In this regard, one potential explanation of the apparent upregulation of phosphopeptides which contain the CSNK2 recognition motif is that phosphopeptides of higher multiplicity (e.g., 2 or 3 phosphorylated residues) may not be phosphorylated by CSNK2 when cells are treated with CX-4945, thereby resulting in an increased abundance of the same peptide of a lower multiplicity. It is often difficult to observe this relationship given the stochastic sampling of abundant peptides by MS; the same peptide of a different multiplicity may not be identified or if it is, it may not be quantified. If this explanation holds true, it underscores the practical limitations of MS in dissecting the phosphoproteome.

## Concluding Remarks and Implications

By using an inhibitor-resistant CSNK2 mutant to validate on-target effects of CX-4945, we devised a strategy for the large-scale identification of CSNK2-dependent substrates in an unbiased, systematic manner. In addition to identifying *bona fide* substrates for CSNK2 that increases our understanding of the role of CSNK2 in cellular signaling, we identified a number of sites which were downregulated in response to CX-4945 treatment due to its off-target effects in cells. This information could help explain the extent to which CX-4945 exhibits its clinical efficacy in the treatment of certain diseases due to CSNK2-dependent inhibition. Considering the promise of CSNK2 as potential therapeutic target, a natural extension of this work could be to exploit this strategy in both normal cells and in cancer cells to reveal the divergence of CSNK2-dependent signaling that accompanies cancer. In a similar respect, we can envisage that the strategy could also be adapted to investigate the relationship between the two closely related isoforms of CSNK2 (ie. CSNK2A1 and CSNK2A2), particularly in terms of determining the extent to which they share overlapping substrates within cells. From this perspective, the need for isoform-specific inhibitors could be overcome by selectively generating inhibitor-resistant mutants of either isoform to elucidate signaling events requiring either CSNK2A1 or CSNK2A2.

Despite having used CX-4945, other inhibitors of CSNK2 could also be employed in this workflow for substrate discovery and characterization of inhibitor potency within cells. For example, during the preparation of this manuscript, a novel CSNK2 inhibitor, SGC-CK2-1, was described as a potent inhibitor of cellular CSNK2 with exceptional specificity *in vitro* (Wells et al., 2021). Although our focus was on CSNK2 substrate elucidation, we also expect that this validation strategy can be adapted for the study of other kinases given the high conservation within the kinase family. Overall, it is of great importance that studies employ a method of validation so that our collective understanding of cellular signalling is not limited by small molecule specificity.

## Figures and Tables

**FIGURE 5:**
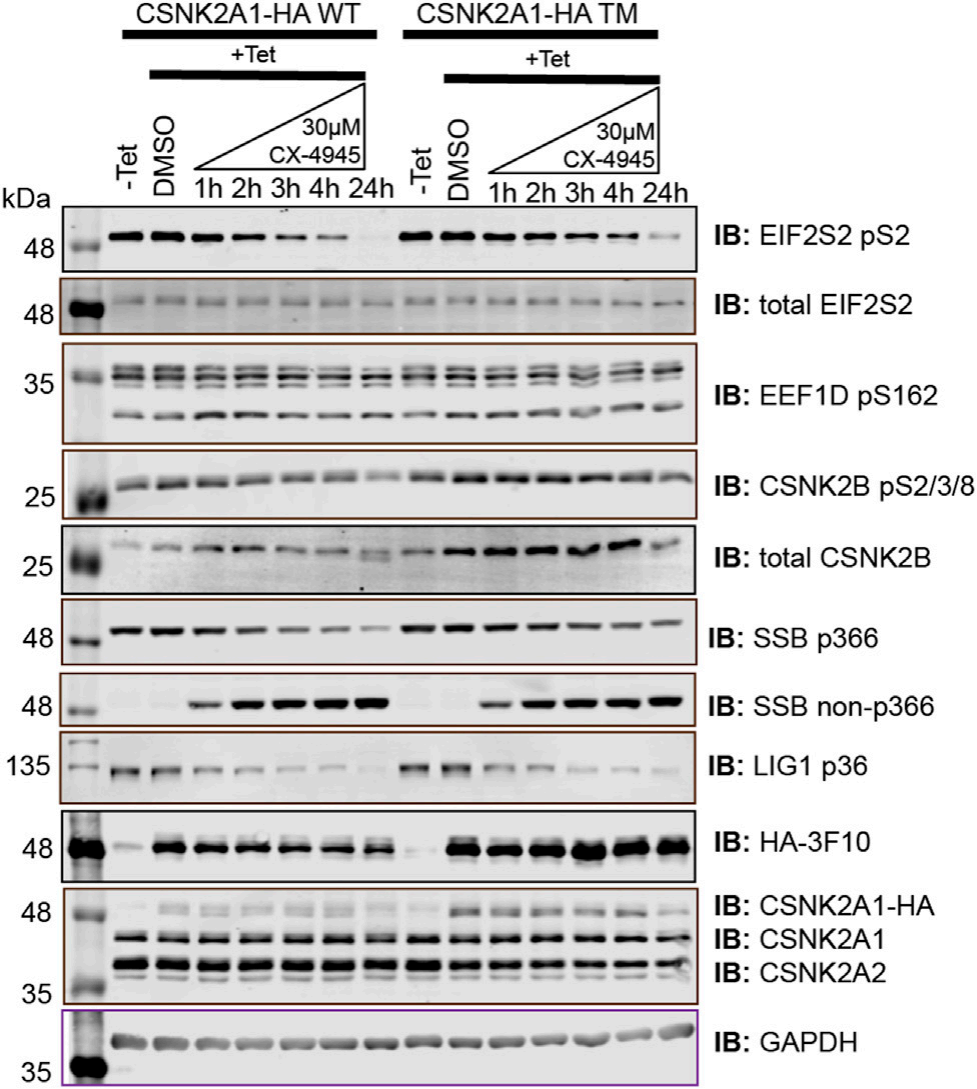
Immunoblot evaluation of phosphospecific antibodies recognizing SSB pS366, SSB S366, and LIG1 pS36. Flp-In T-REx U2OS cell lines stably expressing CSNK2A-HA WT or CSNK2A-HA TM with tight Tetracycline (Tet-ON) regulation were treated with 30 µM of CX-4945 for the indicated times and blotted with the indicated antibodies recognizing total protein or specific sites. Results are representative of two independent experiments.

## Data Availability

The mass spectrometry proteomics data have been deposited to the ProteomeXchange Consortium (Deutsch et al. 2020) via the PRIDE (Perez-Riverol et al., 2022) partner repository with the dataset identifier PXD033523 and 10.6019/PXD0.

## Supplementary Material

Supplementary Figure S1 Comparison of CSNK2 inhibitors for their ability to inhibit cellular CSNK2 with 24 h of treatment at the indicated concentrations in human adenocarcinoma HeLa (HeLaT) cells. DMSO treated lysates +/- λ-phosphatase were used as immunoblotting controls of phospho-specific antibodies. GAPDH was used to demonstrate equal gel loading. Results are representative of two independent experiments.

Supplementary Figure S2 Anti-EIF2S2 pS2 and anti-CSNK2B pS2/3/4/8 antibody (Ab) characterization using HeLaTt cell lysates. Lysate control lanes are treated with +/- λ-phosphatase. The listed EIF2S2 pS2 **(A)** or CSNK2B pS2/3/4/8 (B) peptide sequences were used for phospho-antibody development. The specificity of anti-EIF2S2 pS2 (A) or anti-CSNK2B pS2/3/4/8 **(B)** antibodies was demonstrated in the peptide competition using 200X molar excess of phospho- or nonphospho-peptides. Equal amount of lysate loading was demonstrated with the anti-GAPDH antibody.

Supplementary Figure S3 Anti-SSB pS366, anti-SSB non-phospho S366 and anti-LIG1 pS36 antibody characterization using U2OS cell lysates. Lysate control lanes are treated with +/- λ-phosphatase. The listed SSB pS366 **(A, B)** or LIG1 pS36 **(C)** peptide sequences were used for phospho-antibody development. The specificity of **(A)** anti-SSB pS366 **(B)** anti-SSB non-phospho S366 or **(C)** anti-LIG1 pS36 antibodies is demonstrated in peptide competitions using 200X molar excess of phospho- or nonphospho-peptides. Equal amount of lysate loading was demonstrated with anti-GAPDH antibody.

Supplementary Figure S4 Characterization of Flp-In T-REx U2OS parental, CSNK2A1-HA wild type (WT) and triple mutant (TM, V66A/H160D/I174A) cell lines in immunofluorescence (IF) experiments. Exogenous CSNK2A1-HA WT or CSNK2A1-HA TM expression in the cells were induced with 1 μg/mL Tetracycline. Cells were asynchronous or cell cycle arrested with double Thymidine block (Roberts et al., 2006) before IF labelling with the indicated anti-HA/Alexa 488 (FITC, Thermo Fisher Scientific) antibody and the nucleus was stained with Hoescht 33342 (DAPI, Thermo Fisher Scientific). Scalebar indicates 50 μm on images. Figures are presenting results from two independent experiments.

Supplementary Figure S5 Profile plots of distinct phosphopeptides which were significantly inhibited and rescued, categorized based on corresponding protein. The horizontal dashed line represents 1.5-fold significant inhibition (−0.585).

Supplementary Figure S6 Comparison of rescued phosphopeptides with corresponding differential protein expression. Vertical and horizontal lines (-/+ 0.585) are indicative of significant down- and upregulation of phosphosites and proteins.

Supplementary Figure S7 Profile plots of multiplicity 1 phosphopeptides which were significantly inhibited and partially rescued. Phosphosites with a * represent multiplicity 1 phosphopeptides which satisfied our stringent biological criterion for rescue. The horizontal dashed line represents 1.5-fold significant inhibition (-0.585).

Supplementary Figure S8 CSNK2A1 interaction network. Partially rescued CSNK2 protein substrates and related proteins are depicted in a GeneMANIA network. Previous database information regarding physical interactions (red), co-expression (purple), predicted interactions (orange), genetic interactions (green), co-localization (blue), and shared protein domains (yellow) were employed in network creation in an ‘equal by data type’ weighting approach. Our own list of partially rescued substrates was utilized in network creation and each assigned a value of 1. If a phosphopeptide was not unique and could have originated from several proteins, all potential proteins were assigned a value of 1. Hashed gene names indicate proteins included in our query list, while those with a solid background are related proteins inserted during GeneMANIA network creation. Proteins implicated in Gene Ontology biological processes are labelled.

Supplementary Figure S9 Gene Ontology terms enriched amongst proteins displayed in the GeneMANIA network created using partially rescued protein substrates. Coverage represents the proportion of displayed proteins which are annotated with a certain Gene Ontology term when compared to all genes in the genome annotated with that same term.

## Nomenclature

AKT1: AKT serine/threonine kinase 1
BH: Benjamini-Hochberg
CDC37: Hsp90 co-chaperone Cdc37
CSNK2: casein kinase 2
CSNK2 inhibitor 8: 4-(2-(4-Methoxybenzamido)thiazol-5-yl)benzoic acid
CSNK2A1: Casein kinase II subunit alpha
CSNK2A2: Casein kinase II subunit alpha'
CSNK2B: Casein kinase II subunit beta
CX-4945: 5-(3-chlorophenylamino)benzo[c][2,6]naphthyridine-8-carboxylic acid
DMAT: 2-Dimethylamino-4,5,6,7-tetrabromo-1H-benzimidazole
EEF1D: Elongation factor 1-delta
EIF2S2: Eukaryotic translation initiation factor 2 subunit 2
Ellagic acid: Ellagic acid/Benzoaric acid
FT-U2OS: U2OS cells expressing the tetracycline responsible element of Flp-In™ T-Rex
GO: Gene Ontology
GAPDH: Glyceraldehyde-3-phosphate dehydrogenase
HA: Human influenza hemagglutinin
HeLaT: human adenocarcinoma HeLa Tet-Off (Clontech) expressing the tetracycline responsible element
LIG1: DNA ligase 1
PBST: phosphate-buffered saline with 0.1% Tween 20
Quinalizarin: 1,2,5,8-Tetrahydroxy-9,10-anthraquinone
Resorufin: 7-Hydroxy-3H-phenoxazin-3-one
SILAC: Stable Isotope Labeling by/with Amino acids in Cell culture
SSB: Lupus La protein
TBB: 4,5,6,7-Tetrabromobenzotriazole
TBBz: 4,5,6,7-Tetrabromobenzimidazole
TBST: Tris-buffered saline with 0.05% Tween 20
TM: Triple Mutant (V66A/H160D/I174A) of CSNK2A1-HA with c-term HA tag
U2OS: Human osteosarcoma cell (Tet-Off) expressing tetracycline responsible element
WT: Wild Type
XRCC1: DNA repair protein XRCC1
