# Supplementary material for: Chemical Genetic Validation of CSNK2 Substrates Using an Inhibitor-Resistant Mutant in Combination with Triple SILAC Quantitative Phosphoproteomics
Source: Front Mol Biosci. 2022 Jun 9;9:909711. doi: 10.3389/fmolb.2022.909711 (PMC9225150; doi:10.3389/fmolb.2022.909711)
Supplement: Supplementary file 3 [file DataSheet1.PDF]

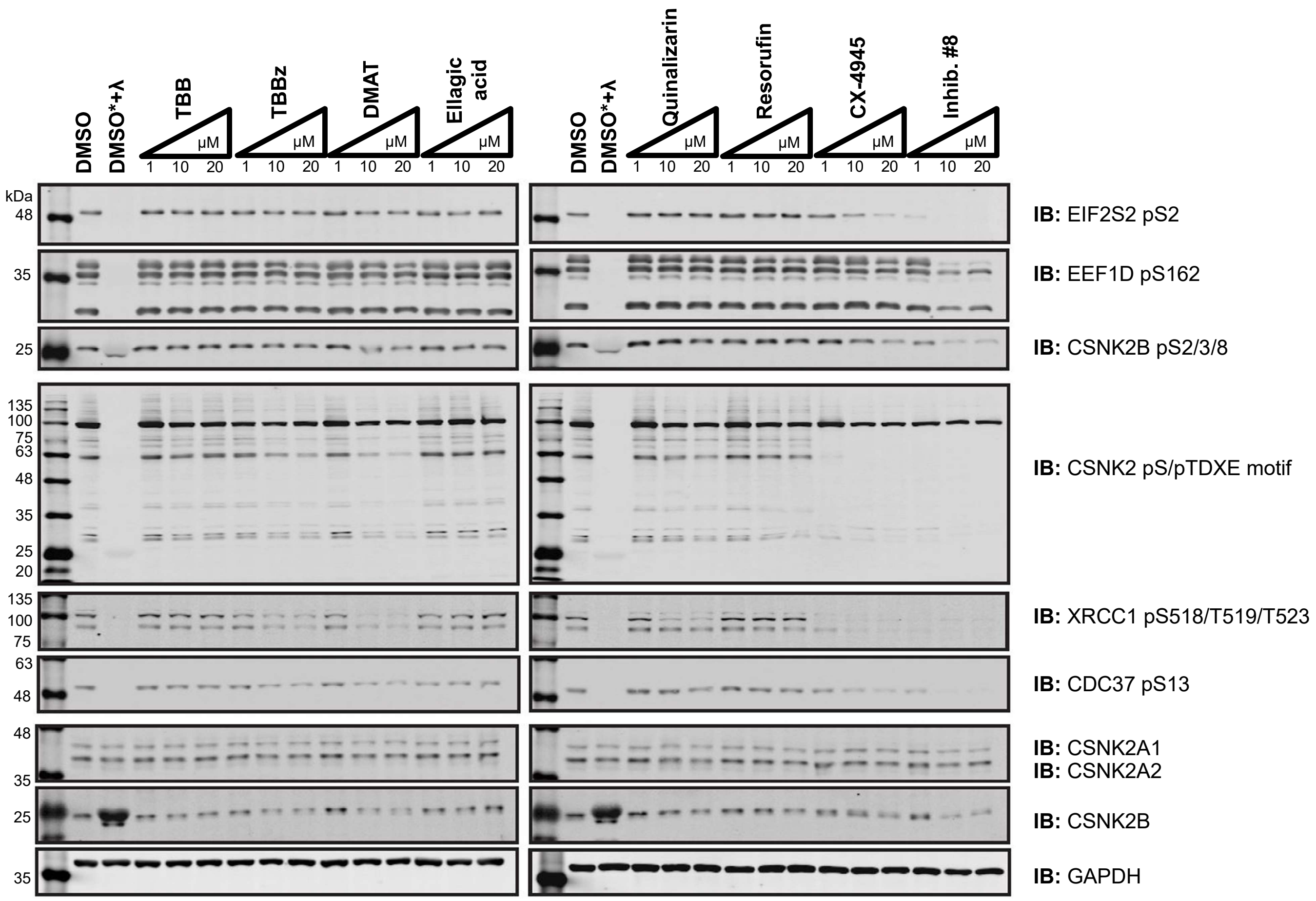
DMSO
DMSO*+λ
TBB
TBBz
DMAT
Ellagic acid
Quinalizarin
Resorufin
CX-4945
Inhib. #8
μM
1 10 20
kDa
IB: EIF2S2 pS2
IB: EEF1D pS162
IB: CSNK2B pS2/3/8
IB: CSNK2 pS/pTDXE motif
IB: XRCC1 pS518/T519/T523
IB: CDC37 pS13
IB: CSNK2A1
IB: CSNK2A2
IB: CSNK2B
IB: GAPDH

Figure S1.

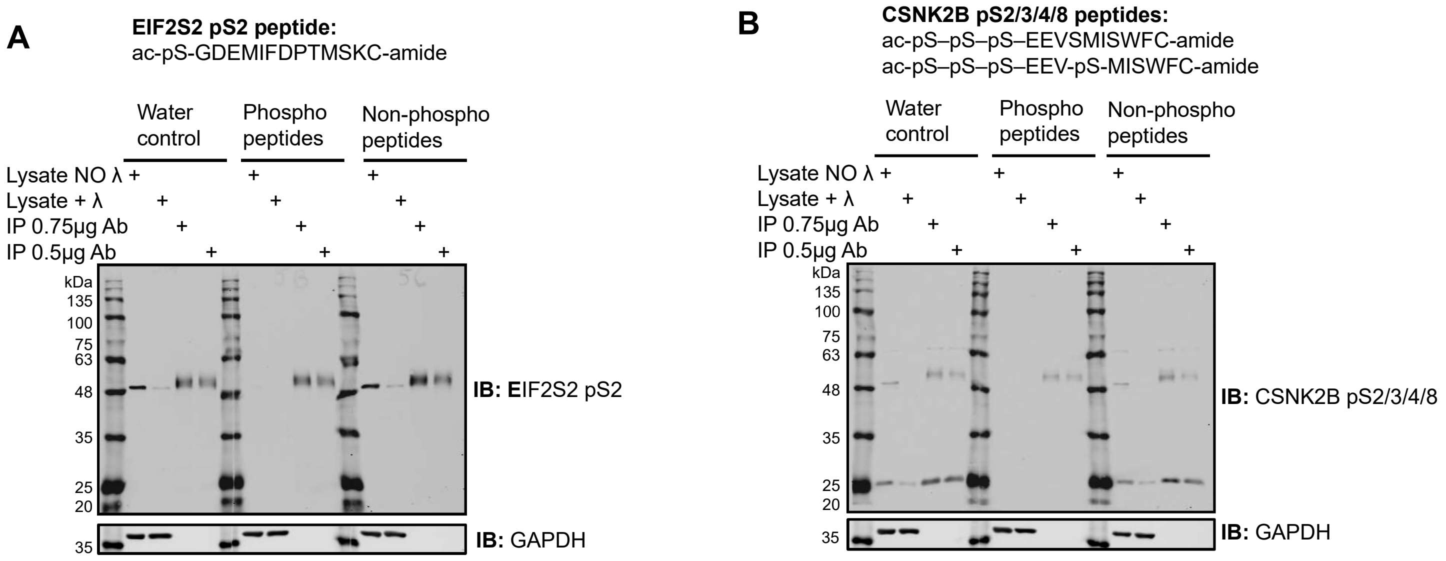
A
EIF2S2 pS2 peptide:
ac-pS-GDEMIFDPTMSKC-amide
Water control
Phospho peptides
Non-phospho peptides
Lysate NO λ
Lysate + λ
IP 0.75µg Ab
IP 0.5µg Ab
kDa
135
100
75
63
48
35
25
20
IB: EIF2S2 pS2
35
IB: GAPDH
B
CSNK2B pS2/3/4/8 peptides:
ac-pS–pS–pS–EEVSMISWFC-amide
ac-pS–pS–pS–EEV-pS-MISWFC-amide
Water control
Phospho peptides
Non-phospho peptides
Lysate NO λ
Lysate + λ
IP 0.75µg Ab
IP 0.5µg Ab
kDa
135
100
75
63
48
35
25
20
IB: CSNK2B pS2/3/4/8
35
IB: GAPDH

Figure S2.

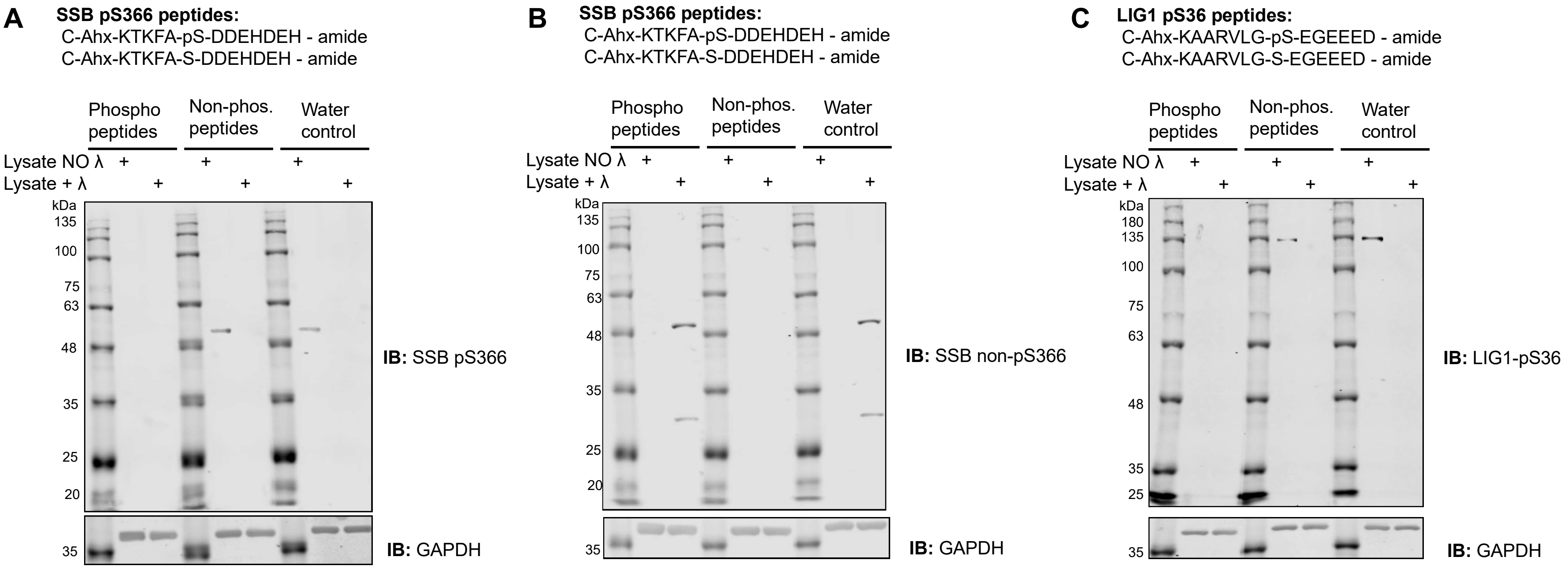
A
SSB pS366 peptides:
C-Ahx-KTKFA-pS-DDEHDEH - amide
C-Ahx-KTKFA-S-DDEHDEH - amide
Phospho peptides
Non-phos. peptides
Water control
Lysate NO λ
Lysate + λ
kDa
135
100
75
63
48
35
25
20
IB: SSB pS366
35
IB: GAPDH
B
SSB pS366 peptides:
C-Ahx-KTKFA-pS-DDEHDEH - amide
C-Ahx-KTKFA-S-DDEHDEH - amide
Phospho peptides
Non-phos. peptides
Water control
Lysate NO λ
Lysate + λ
kDa
135
100
75
63
48
35
25
20
IB: SSB non-pS366
35
IB: GAPDH
C
LIG1 pS36 peptides:
C-Ahx-KAARVLG-pS-EGEEED - amide
C-Ahx-KAARVLG-S-EGEEED - amide
Phospho peptides
Non-phos. peptides
Water control
Lysate NO λ
Lysate + λ
kDa
180
135
100
75
63
48
35
25
IB: LIG1-pS36
35
IB: GAPDH

Figure S3.

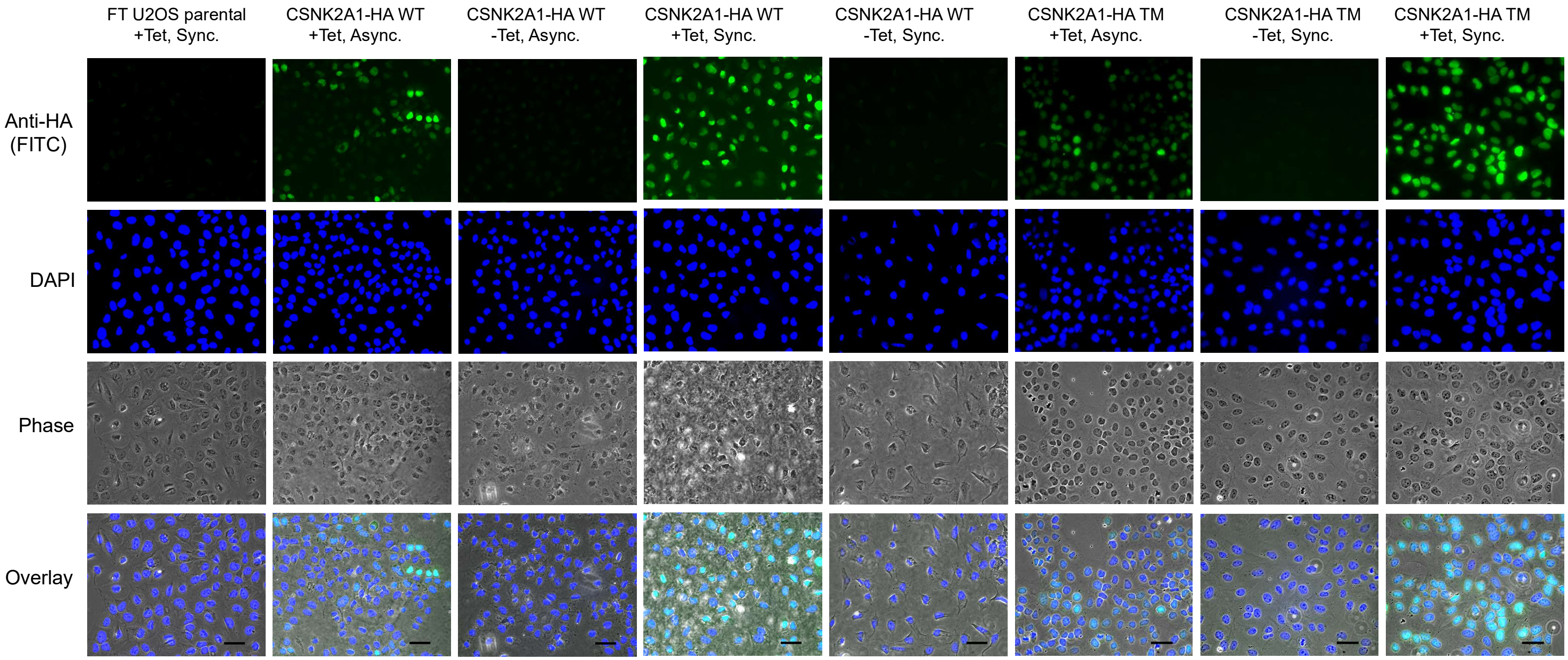
FT U2OS parental +Tet, Sync.
CSNK2A1-HA WT +Tet, Async.
CSNK2A1-HA WT -Tet, Async.
CSNK2A1-HA WT +Tet, Sync.
CSNK2A1-HA WT -Tet, Sync.
CSNK2A1-HA TM +Tet, Async.
CSNK2A1-HA TM -Tet, Sync.
CSNK2A1-HA TM +Tet, Sync.
Anti-HA (FITC)
DAPI
Phase
Overlay

Figure S4.

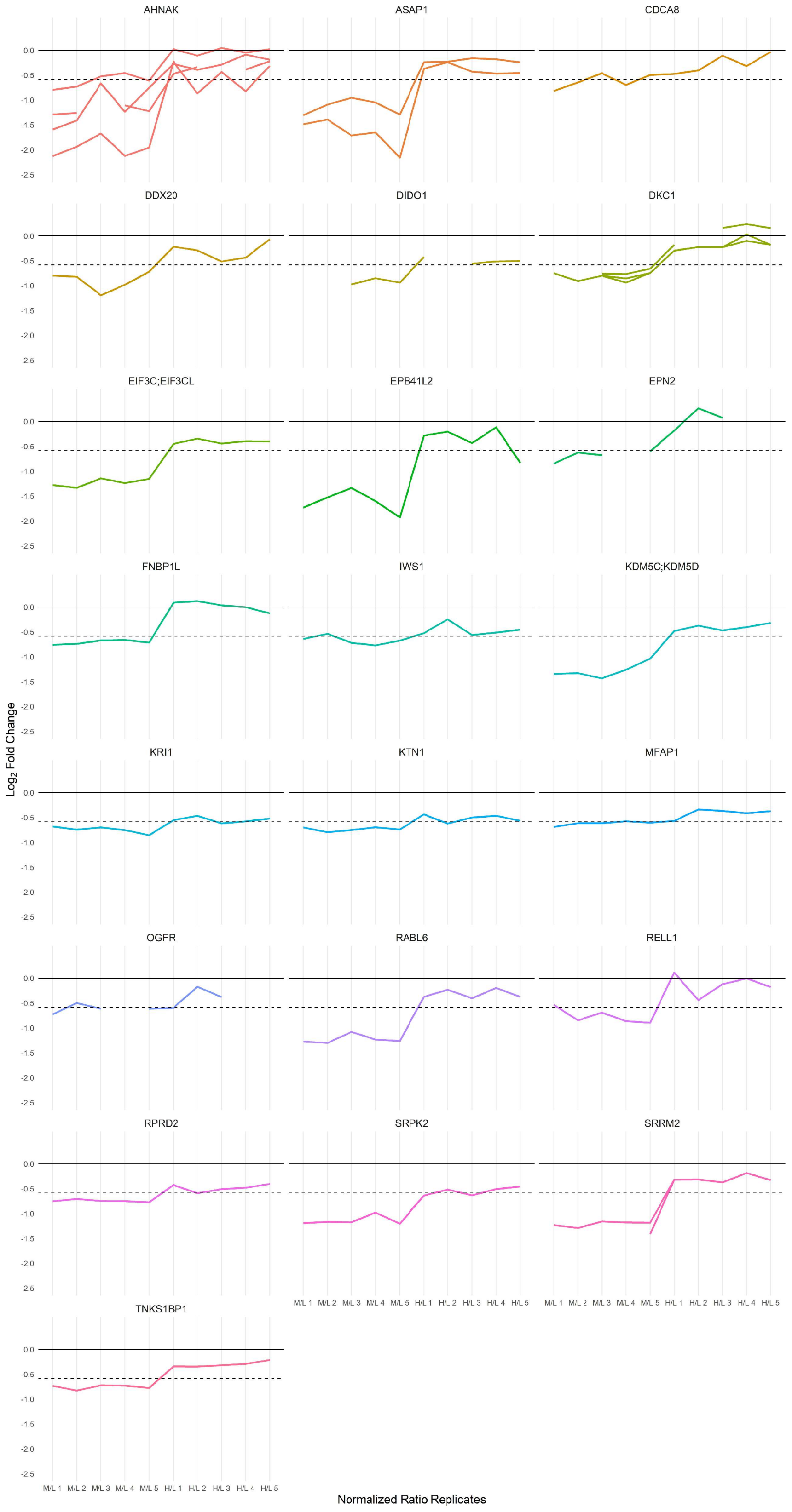
AHNAK
ASAP1
CDCA8
DDX20
DIDO1
DKC1
EIF3C;EIF3CL
EPB41L2
EFN2
FNBP1L
IWS1
KDM5C;KDM5D
KRI1
KTN1
MFAP1
OGFR
RABL6
RELL1
RPRD2
SRPK2
SRRM2
TNKS1BP1
0.0
-0.5
-1.0
-1.5
-2.0
-2.5
Log2 Fold Change
M/L 1
M/L 2
M/L 3
M/L 4
M/L 5
H/L 1
H/L 2
H/L 3
H/L 4
H/L 5
Normalized Ratio Replicates

Figure S5.

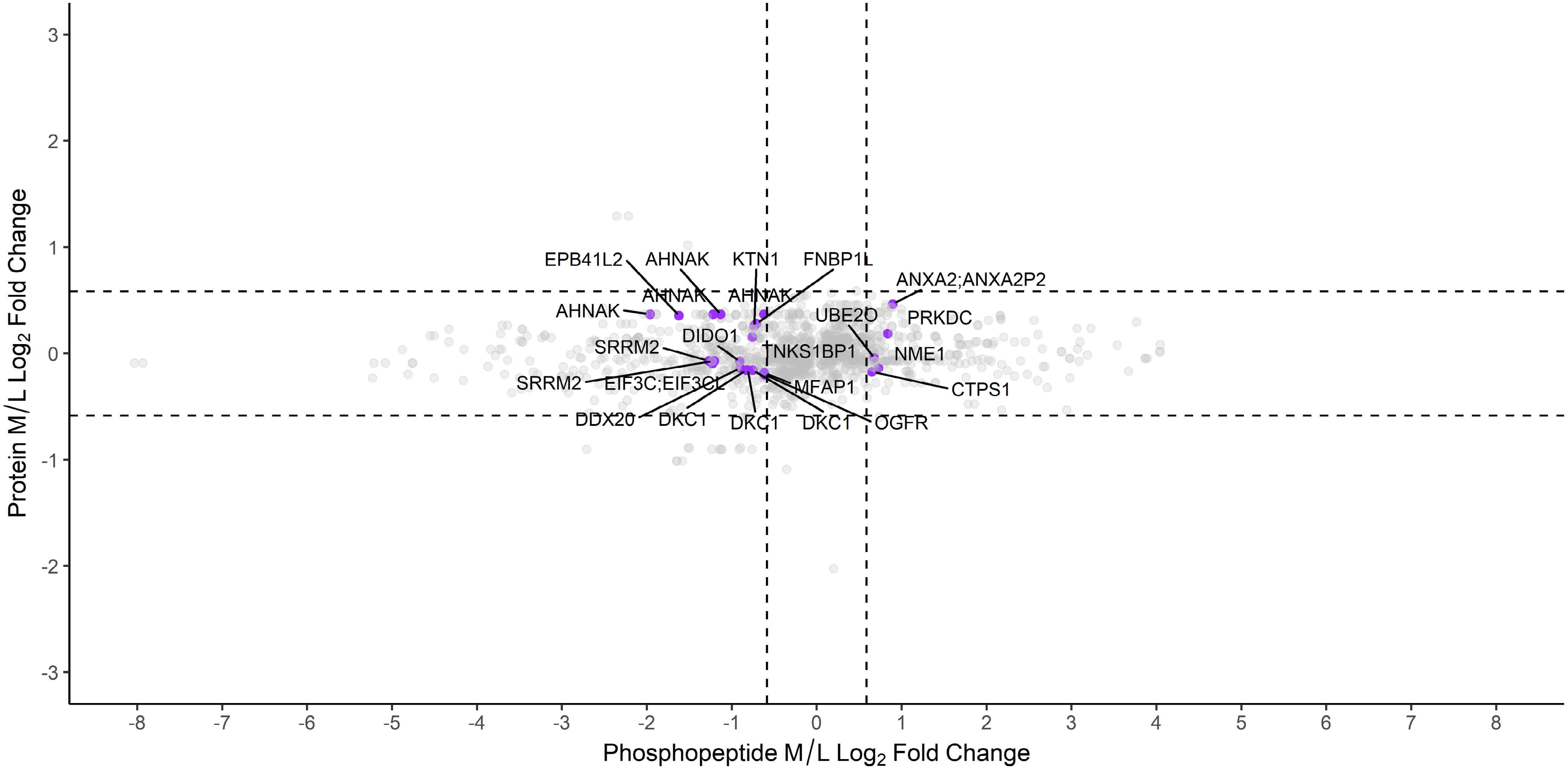

Protein M/L Log2 Fold Change
Phosphopeptide M/L Log2 Fold Change
3
2
1
0
-1
-2
-3
-8
-7
-6
-5
-4
-3
-2
-1
0
1
2
3
4
5
6
7
8
EPB41L2
AHNAK
KTN1
FNBP1L
ANXA2;ANXA2P2
AHNAK
AHNAK
AHNAK
UBE2O
PRKDC
DIDO1
SRRM2
NKS1BP1
NME1
SRRM2
EIF3C;EIF3CL
MFAP1
CTPS1
DDX20
DKC1
DKC1
DKC1
OGFR

Figure S6.

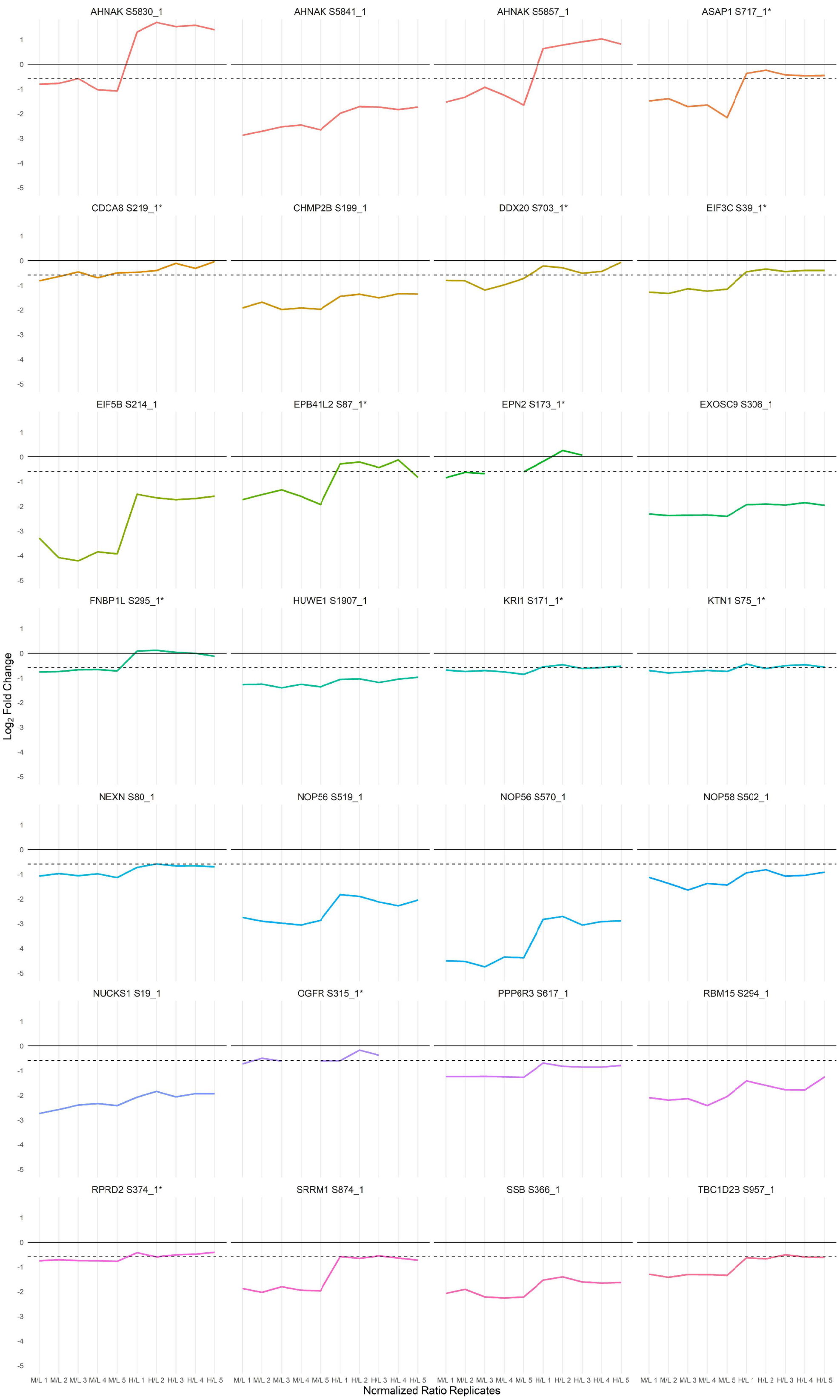
AHNAK S5830_1
AHNAK S5841_1
AHNAK S5857_1
ASAP1 S717_1*
CDCA8 S219_1*
CHMP2B S199_1
DDX20 S703_1*
EIF3C S39_1*
EIF5B S214_1
EPB41L2 S87_1*
EPN2 S173_1*
EXOSC9 S306_1
FNBP1L S295_1*
HUWE1 S1907_1
KRI1 S171_1*
KTN1 S75_1*
NEXN S80_1
NOP56 S519_1
NOP56 S570_1
NOP58 S502_1
NUCKS1 S19_1
OGFR S315_1*
PPP6R3 S617_1
RBM15 S294_1
RPRD2 S374_1*
SRRM1 S874_1
SSB S366_1
TBC1D2B S957_1
Log2 Fold Change
M/L 1 M/L 2 M/L 3 M/L 4 M/L 5 H/L 1 H/L 2 H/L 3 H/L 4 H/L 5
Normalized Ratio Replicates

Figure S7.

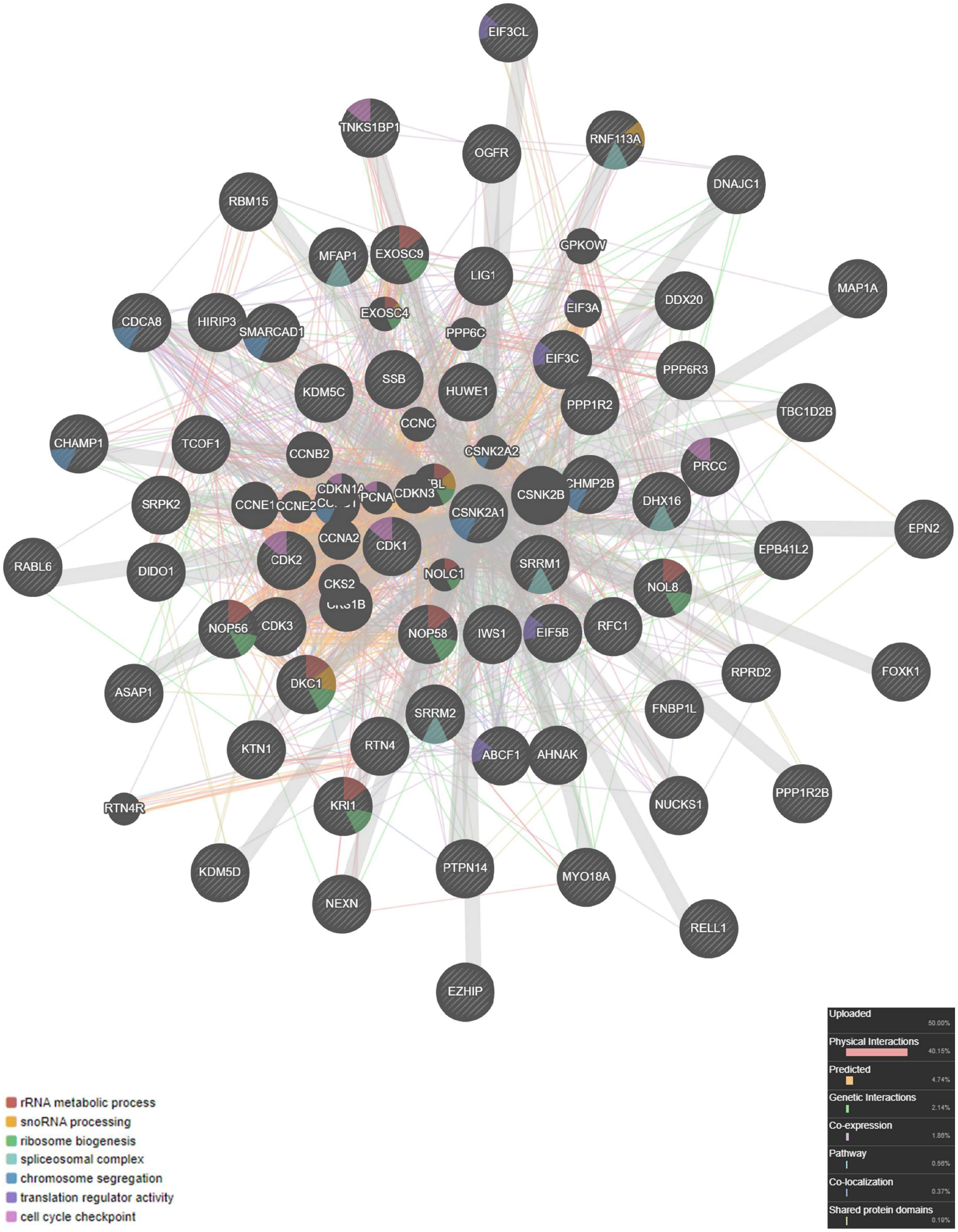
EIF3CL
TNKS1BP1
OGFR
RNF113A
DNAJC1
RBM15
MFAP1
EXOSC9
GPKOW
LIG1
MAP1A
EIF3A
DDX20
CDCA8
HIRIP3
SMARCAD1
EXOSC4
PPP6C
EIF3C
PPP6R3
SSB
KDM5C
HUWE1
PPP1R2
TBC1D2B
CCNC
CHAMP1
TCOF1
CCNB2
CSNK2A2
PRCC
CDKN1A
PCNA
CDKN3
CSNK2B
CHMP2B
DHX16
SRPK2
CCNE1
CCNE2
CSNK2A1
EPN2
CCNA2
CDK1
EPB41L2
RABL6
DIDO1
CDK2
SRRM1
NOLC1
NOL8
CKS2
NOP56
CDK3
NOP58
IWS1
EIF5B
RFC1
RPRD2
FOXK1
ASAP1
DKC1
FNBP1L
SRRM2
KTN1
RTN4
ABCF1
AHNAK
RTN4R
KRI1
NUCKS1
PPP1R2B
KDM5D
PTPN14
MYO18A
NEXN
RELL1
EZHIP
rRNA metabolic process
snoRNA processing
ribosome biogenesis
spliceosomal complex
chromosome segregation
translation regulator activity
cell cycle checkpoint
Uploaded
50.00%
Physical Interactions
40.15%
Predicted
4.74%
Genetic Interactions
2.14%
Co-expression
1.86%
Pathway
0.56%
Co-localization
0.37%
Shared protein domains
0.19%

Figure S8.

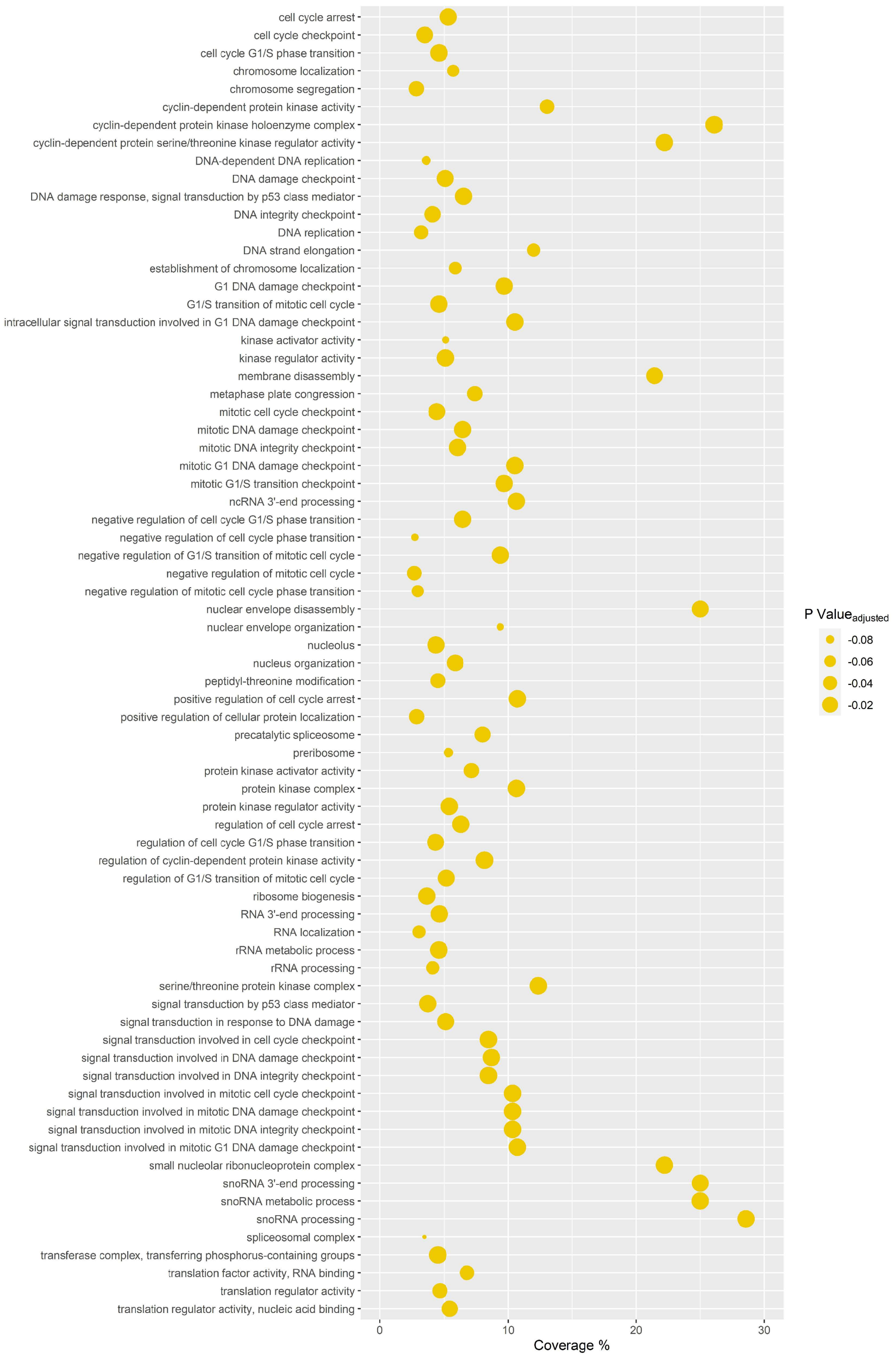
cell cycle arrest
cell cycle checkpoint
cell cycle G1/S phase transition
chromosome localization
chromosome segregation
cyclin-dependent protein kinase activity
cyclin-dependent protein kinase holoenzyme complex
cyclin-dependent protein serine/threonine kinase regulator activity
DNA-dependent DNA replication
DNA damage checkpoint
DNA damage response, signal transduction by p53 class mediator
DNA integrity checkpoint
DNA replication
DNA strand elongation
establishment of chromosome localization
G1 DNA damage checkpoint
G1/S transition of mitotic cell cycle
intracellular signal transduction involved in G1 DNA damage checkpoint
kinase activator activity
kinase regulator activity
membrane disassembly
metaphase plate congression
mitotic cell cycle checkpoint
mitotic DNA damage checkpoint
mitotic DNA integrity checkpoint
mitotic G1 DNA damage checkpoint
mitotic G1/S transition checkpoint
ncRNA 3'-end processing
negative regulation of cell cycle G1/S phase transition
negative regulation of cell cycle phase transition
negative regulation of G1/S transition of mitotic cell cycle
negative regulation of mitotic cell cycle
negative regulation of mitotic cell cycle phase transition
nuclear envelope disassembly
nuclear envelope organization
nucleolus
nucleus organization
peptidyl-threonine modification
positive regulation of cell cycle arrest
positive regulation of cellular protein localization
precatalytic spliceosome
preribosome
protein kinase activator activity
protein kinase complex
protein kinase regulator activity
regulation of cell cycle arrest
regulation of cell cycle G1/S phase transition
regulation of cyclin-dependent protein kinase activity
regulation of G1/S transition of mitotic cell cycle
ribosome biogenesis
RNA 3'-end processing
RNA localization
rRNA metabolic process
rRNA processing
serine/threonine protein kinase complex
signal transduction by p53 class mediator
signal transduction in response to DNA damage
signal transduction involved in cell cycle checkpoint
signal transduction involved in DNA damage checkpoint
signal transduction involved in DNA integrity checkpoint
signal transduction involved in mitotic cell cycle checkpoint
signal transduction involved in mitotic DNA damage checkpoint
signal transduction involved in mitotic DNA integrity checkpoint
signal transduction involved in mitotic G1 DNA damage checkpoint
small nucleolar ribonucleoprotein complex
snoRNA 3'-end processing
snoRNA metabolic process
snoRNA processing
spliceosomal complex
transferase complex, transferring phosphorus-containing groups
translation factor activity, RNA binding
translation regulator activity
translation regulator activity, nucleic acid binding
0
10
20
30
Coverage %
P Value adjusted
-0.08
-0.06
-0.04
-0.02

Figure S9.
